# A Systematic Study of Yiqi Qubai Standard Decoction for Treating Vitiligo Based on UPLC-Q-TOF/MS Combined with Chemometrics, Molecular Docking, and Cellular and Zebrafish Assays

**DOI:** 10.3390/ph16121716

**Published:** 2023-12-11

**Authors:** Lijun Cui, Cui Ma, Wenqing Shi, Chen Yang, Jiangping Wu, Zhenghua Wu, Yuefen Lou, Guorong Fan

**Affiliations:** 1 School of Medicine, Tongji University, Shanghai 200331, China; cuilijun620@163.com; 2 School of Pharmacy, Naval Medical University, Shanghai 200433, China; 3 Department of Clinical Pharmacy, Shanghai General Hospital, Shanghai Jiaotong University School of Medicine, Shanghai 200080, Chinachenyang9709040731@163.com (C.Y.); wujiangping@whu.edu.cn (J.W.); wuzhenghua526@163.com (Z.W.); 4 School of Pharmacy, Shanghai Jiaotong University, Shanghai 200240, China; 5 Department of Pharmacy, Shanghai Fourth People’s Hospital Affiliated to Tongji University School of Medicine, Shanghai 200434, China; 6 School of Pharmacy, Anhui University of Chinese Medicine, Hefei 230012, China

**Keywords:** Yiqi Qubai standard decoction, vitiligo, UPLC-Q-TOF/MS, chemometrics, network pharmacological, molecular docking, melanogenesis, B16F10 cells, zebrafish

## Abstract

The Yiqi Qubai (YQ) formula is a hospital preparation for treating vitiligo in China that has had reliable efficacy for decades. The formula consists of four herbs; however, the extraction process to produce the formula is obsolete and the active ingredients and mechanisms remain unknown. Therefore, in this paper, fingerprints were combined with the chemometrics method to screen high-quality herbs for the preparation of the YQ standard decoction (YQD). Then, the YQD preparation procedure was optimized using response surface methodology. A total of 44 chemical constituents, as well as 36 absorption components (in rat plasma) of YQD, were identified via UPLC-Q-TOF/MS. Based on the ingredients, the quality control system of YQD was optimized by establishing the SPE-UPLC-Q-TOF/MS identification method and the HPLC quantification method. Network pharmacological analysis and molecular docking showed that carasinaurone, calycosin-7-*O*-β-d-glucoside, methylnissolin-3-*O*-glucoside, genkwanin, akebia saponin D, formononetin, akebia saponin B, and apigenin may be the key active components for treating vitiligo; the core targets associated with them were AKT1, MAPK1, and mTOR, whereas the related pathways were the PI3K-Akt, MAPK, and FoxO signaling pathways. Cellular assays showed that YQD could promote melanogenesis and tyrosinase activity, as well as the transcription and expression of tyrosinase-associated proteins (i.e., TRP-1) in B16F10 cells. In addition, YQD also increased extracellular tyrosinase activity. Further efficacy validation showed that YQD significantly promotes melanin production in zebrafish. These may be the mechanisms by which YQD improves the symptoms of vitiligo. This is the first systematic study of the YQ formula that has optimized the standard decoction preparation method and investigated the active ingredients, quality control, efficacy, and mechanisms of YQD. The results of this study lay the foundations for the clinical application and further development of the YQ formula.

## 1. Introduction

Vitiligo is the most common hypopigmentation disorder. It is an acquired disease characterized by progressive loss of melanocytes. Its global prevalence is 0.5–2%, with no racial or sex differences [1]. Vitiligo is a complex disease whose pathogenesis remains unknown but may be related to genetic variation, autoimmunity, oxidative stress, the autophagy of melanocytes, and/or neuroendocrine function [2]. The goal of vitiligo treatment is to suppress depigmentation and stimulate repigmentation in the patients. Currently, the available treatments for vitiligo mainly include glucocorticoids, immunosuppressants, phototherapy, and surgical methods [3,4,5,6]. Unfortunately, the available therapies are not effective for all patients, and some have wide-ranging side effects that suppress further treatment [7]. Therefore, increasing research has focused on finding new multi-target therapeutic medicine with fewer side effects to fit the complex treatment mechanisms of vitiligo and the need for long-term medication. 

There is a long history of using traditional Chinese medicine (TCM) to treat vitiligo in China, with which there have been positive results and few adverse effects [8]. The theory of using TCM in the treatment of vitiligo is that it benefits “qi” by activating “blood” and dispelling pathologic “wind” [9]. “Qi” is a theory of Chinese medicine that represents the substance that constitutes the human body, maintains human life activities, and promotes Yang, the driving force of metabolism and physiological function of the human body [10]. The Yiqi Qubai (YQ) formula is a TCM that has been utilized in the dermatology department of Shanghai General Hospital for decades [11] and has had remarkable curative results [12,13]. It consists of four herbs, *Astragali radix* (Huangqi, HQ), *Akebiae fructus* (Yuzhizi, YZZ), *Leonuri fructus* (Chongweizi, CWZ), and *Caragana sinica roots* (Jinquegen, JQG), in a weight ratio of 1:1:0.75:1.5. Each of the four herbs can soothe the liver and regulate “qi”, activate “blood”, and dispel “wind” [14,15,16]. The upregulation of mushroom tyrosinase (TYR) activity and melanogenesis of YQ was demonstrated using serum pharmacological methods [17]. Although YQ has been used clinically for many years, there is a lack of systematic research on it. Firstly, the decoction process does not follow the standard decoction principles, which may affect the uniformity of the preparation quality. Secondly, the composition of the decoction is unclear, which is not conducive to an in-depth study of its material basis and mechanisms. In addition, the identification methods in the existing quality standards are simple and lack quantitative methods to ensure quality. 

Essentially, modern TCM preparations rely on the decoction, which is the oldest Chinese medicinal compound that can reflect the material basis of a prescription; therefore, the quality of the decoction is crucial. The Chinese Pharmacopoeia Commission formally proposed the “standard decoction” in 2016, which is characterized by the authenticity of the herbs, the uniformity of the extraction process, and the rigor of quality control [18]. The preparation principle of the standard decoction is based on the theory of TCM, following the boiling principle of TCM decoction, adopting standardized technology, and decoction with water. The preparation method is recommended to be optimized with reference to “the management standard of traditional Chinese medicine decoctions in medical institutions” published by the State Drug Administration of China [19]. The standard decoction of TCMs has a uniform process and a product with consistent quality; this will assist in the preparation of granules and allow preparations to be widely used in clinical practice. In order to ensure the accuracy of the medication and the consistency of the administered dose, it is necessary to establish a method for the preparation of a YQ standard decoction (YQD) that could be the basis for the clinical use of the medication.

As the raw material of a standard decoction, the quality of the TCM herbs is affected by origin, harvesting season, storage time, processing method, etc., which largely determines the efficacy of TCM preparations. The TCM fingerprint is a comprehensive, quantifiable technology that can provide more detailed information regarding the type and contents of the TCM components [20,21]. As data mining methods have developed, chemometrics has gained more and more attention because it can simplify complex data and uncover hidden patterns. The combination of chemometric methods such as hierarchical cluster analysis (HCA) and principal component analysis (PCA) with fingerprints is often used to classify and evaluate the quality of TCM herbs [22,23]. 

In recent years, mouse B16F10 melanoma cells have been used as a sensitive and reliable model for the quantitative analysis of melanin [24]. In addition, zebrafish have a high degree of chromosome homology with humans and have a translucent body that allows direct visualization of the development of melanin streaks, making it a suitable model organism for studying melanogenesis in vivo [25]. Melanocyte-specific enzymes, such as TYR and the TYR-associated proteins TRP-1 and TRP-2, are involved in melanin synthesis. TYR is the main rate-limiting enzyme in melanin synthesis, and any factor that activates TYR activity can effectively promote the production of melanin [26].

**Figure 1:**
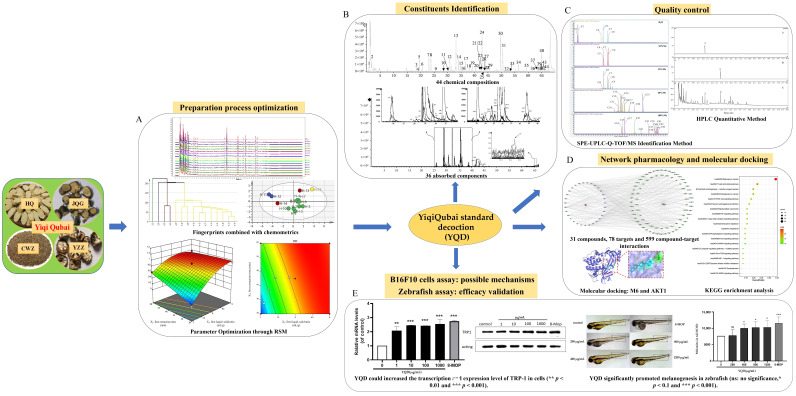
Schematic diagram of the study process.

In this study, HPLC fingerprints were combined with chemometrics to establish a quality control system of raw herbs to screen high-quality herbs for the preparation of YQD. The preparation process of YQD was then optimized via response surface methodology (RSM). A UPLC-Q-TOF/MS technique was used to analyze the chemical constituents of YQD and the absorption components (in rat plasma). Next, the SPE-UPLC-Q-TOF/MS identification method and HPLC quantification method were developed to improve the quality control system. Network pharmacology analysis and molecular docking based on the identified components were used to predict the active ingredients, targets, and mechanisms of vitiligo treatment. Then, we investigated the effects of YQD on melanogenesis, TYR activity, and TRP-1 expression in B16F10 cells, as well as extracellular TYR activity, to explore the possible mechanisms. In addition, the efficacy was investigated by studying its effect on melanogenesis in zebrafish. To the best of our knowledge, this is the first systematic study on the preparation, composition, quality control, efficacy, and mechanism of YQD. The results of this study lay the foundations for the clinical application and further development of YQD. The whole study process is summarized in Figure 1.

## 2. Results and Discussion

### 2.1. Multivariate Statistical Analysis of the HPLC Fingerprint in Raw Herbs

#### 2.1.1. HPLC-DAD Analysis and Similarity Evaluation

**Figure 2:**
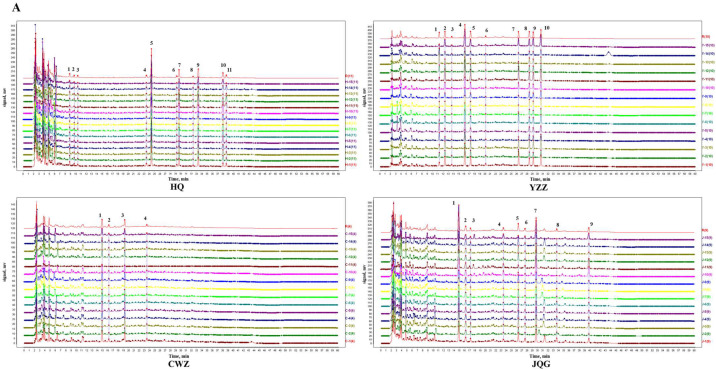

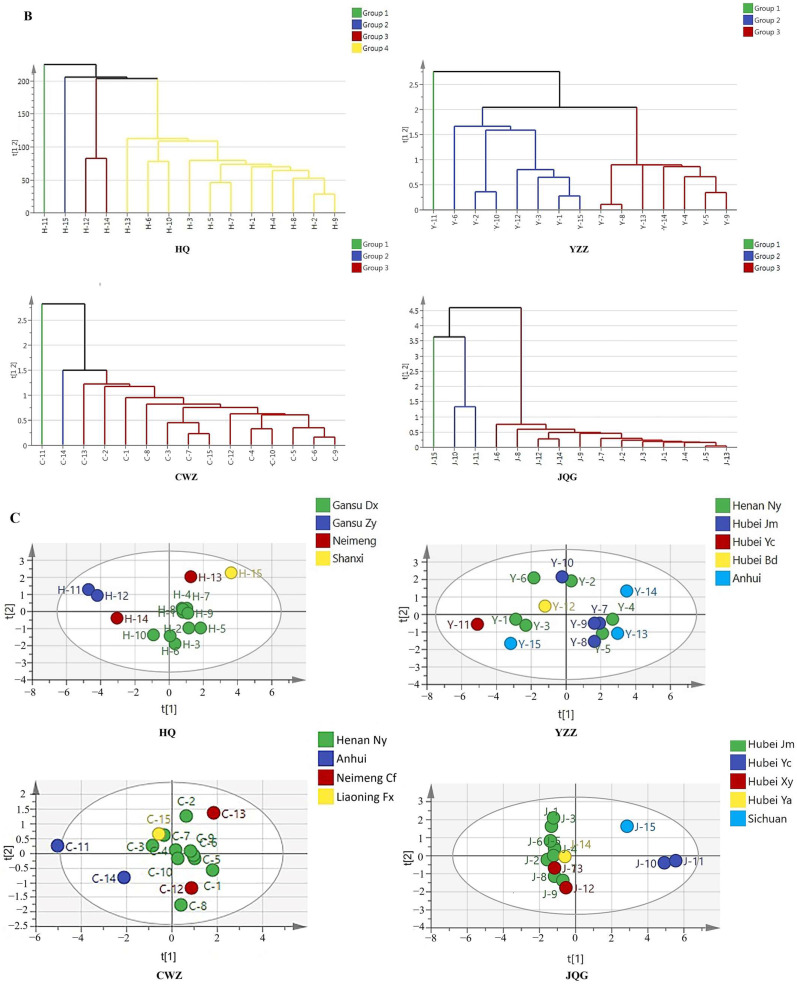
Statistical analysis based on the fingerprints of 15 batches of raw herbs. (**A**) Similarity matching of HPLC fingerprints (numbers represent the common peaks); (**B**) results of the hierarchical cluster analysis (HCA); and (**C**) principal component analysis (PCA) score scatters (HQ: Huangqi; YZZ: Yuzhizi; CWZ: Chongweizi; JQG: Jinquegen).

Biological and abiotic factors affected the chemical quality of plants and showed wide geographical variation. In order to ensure the stability of the raw materials, it is necessary to study the origins before establishing a standard decoction preparation process. Using HPLC-DAD, 15 batches of HQ (H-1–H-15), YZZ (Y-1–Y-15), CWZ (C-1–C-15), and JQG (J-1–J-15) from different origins were detected and their chromatograms were recorded. The precision, repeatability, and stability of the analytical method were validated. The results indicated that the fingerprint analysis method was stable and reliable. The similarity of the fingerprint chromatograms was assessed. Table S1 shows the similarity matching results for the fingerprints. A total of 11, 10, 4, and 9 common peaks were identified on the fingerprint spectra for HQ, YZZ, CWZ, and JQG, respectively (Figure 2A). The intragroup similarities of HQ, YZZ, CWZ, and JQG were 0.919–0.998, 0.773–0.997, 0.932–0.999, and 0.883–0.999, respectively. As shown in the results, each of the four herbs, when obtained from different production areas, differed within the group, especially YZZ and JQG; these differences may be due to the different geographies, harvesting times, and sources of the herbs.

#### 2.1.2. Hierarchical Cluster Analysis (HCA)

In order to evaluate the similarity of the samples based on their fingerprint data, HCA was used to categorize them [22]. As shown in Figure 2B, HQ samples were divided into four clusters, of which H-11 (Zhangye, Gansu) and H-15 (Shanxi) were classified as group I and group II, respectively, H-12 (Zhangye, Gansu) and H-14 (Neimeng) were classified as group III, and the remaining samples (Dingxi, Gansu) were classified as group IV. The YZZ samples were divided into three clusters, of which Y-11 (Yichang, Hubei) was a single group. CWZ samples were divided into three clusters, C-11 (Nanyang, Henan) and C-14 (Anhui) were classified as group I and group II, respectively, and the other samples (Nanyang, Henan; Chifeng, Neimeng; and Fuxin, Liaoning) were classified as group III. The JQG samples were divided into three clusters, J-15 (Sichuan) was alone in group I, J-10 (Yichang, Hubei) and J-11 (Yichang, Hubei) were in group II, and the remaining samples (Jingmen/Xiangyang/Yuan’an, Hubei) were classified as group III. The findings reveal that samples from similar origins were more likely to be placed into the same group, meaning the content of the ingredients was comparable. Nevertheless, some samples from the same origin were categorized independently, probably because of batch variations. There was not a clear regional clustering of YZZ, which may be related to the fact that it is collected from a variety of sources (YZZ is the dry fruit of *Akebia quinata* (Thunb.) Decne., *A. trifoliata* (Thunb.) Koidz. var. *australis* (Diels) Rehd., and *A. trifoliata* (Thunb.) Koidz.). Differences in the chemical makeup of the herbs can affect their quality; therefore, the quality of herbs from various locations needs to be assessed to determine the best source of raw materials.

#### 2.1.3. Principal Component Analysis (PCA)

As a statistical method of dimension reduction, PCA recombines the original variables into independent comprehensive variables [23]. Calculating the composite score of principal components (PCs) and sorting could help to determine the relative quality of each sample. In this work, PCA was performed using SIMCA (ver. 14.1). The eigenvalue and variance contribution rate of the correlation coefficient are shown in Table S2. With an eigenvalue >0.99, the first three PCs in HQ had the cumulative variance contribution rate of 81.609%; the first two PCs in YZZ had the cumulative variance contribution rate of 85.467%; the first one PC in CWZ was 73.803%; and the first three PCs in JQG had the cumulative variance contribution rate of 88.242%. These PCs contained the most information on the variables and fully reflected the original data. The factor load matrix and loadings plot (Figure S1) revealed that peaks 5 (calycosin-7-O-D-glucoside), 7, 8, and 11 in HQ; peaks 5 (cryptochlorogenic acid), 4, 7, and 2 in YZZ; peaks 1, 3, and 2 (chlorogenic acid) in CWZ; and peaks 7, 1, and 9 in JQG all demonstrated a higher contribution to PC1 than the other peaks. These peaks might be the primary chemical characteristic peaks that can be utilized to differentiate the quality of the different varieties of the four herbs.

The PC scores and comprehensive score values of the 15 batches of HQ, YZZ, CWZ, and JQG were calculated using the initial eigenvalue and weights of the PCs. A higher score means better quality. As shown in Table 1, the comprehensive score of H-12 (Zhangye, Gansu), Y-11 (Yichang, Hubei), C-13 (Chifeng, Neimeng), and J-11 (Yichang, Hubei) were highest for the HQ, YZZ, CWZ, and JQG samples, respectively. Similar to the HCA, samples with the highest and lowest comprehensive scores were individually clustered in the PCA score chart (Figure 2C). The ecological settings and herb quality are closely connected, and standard decoctions should be made from suitable and stable herbs. Our results showed that H-12, Y-11, C-13, and J-11 were better quality and should be used for the subsequent preparation of YQD.

### 2.2. Optimization of YQD Preparation Process

#### 2.2.1. Parameter Optimization through RSM

RSM is a collection of statistical and mathematical methodologies for developing and optimizing procedures in which many variables impact a desired response; it has the aim of optimizing that response [27]. The Box–Behnken design (BBD), a type of RSM, reduces the number of tests and investigates the interactions between the influencing factors to obtain the optimal process parameters quickly and effectively [28]. The experimental parameters were optimized using BBD based on the results of the single-factor studies of the solid–liquid ratio, decoction time, and concentration process. Three independent variables, namely, the first solid–liquid ratio (X_1_), the first extraction time (X_2_), and the second extraction time (X_3_), were selected to be included in the response surface modeling, and a three-factor, three-level (−1, 0, +1) BBD was carried out. Synthesizing all indicators (calycosin-7-*O*-β-d-glucoside and chlorogenic acid contents of the extract and its total solid content), the overall desirability (OD) value was used to express the overall impact results. According to the Hassan method [29], the OD value was calculated as follows:

di = (Yi − Ymin)/(Ymax − Ymin)(1)

The di value of each indicator was used to calculate the total OD as follows [30]:

OD = (d_1_ × d_2_…dx) ^1/*n*^ (2)

A total of 17 experiments were conducted (*n* = 17) and the BBD test design and response values are shown in Table 2. To determine the predicted response values, the following equations were fitted using a quadratic mode:

OD = 0.8612 + 0.3228X_1_ + 0.1158X_2_ + 0.1248X_3_ − 0.1440X_1_X_2_ − 0.1435X_1_X_3_ − 0.0205X_2_X_3_ − 0.2179X_1_^2^ − 0.0159X_2_^2^ − 0.0058X_3_^2^

where X_1_ is the first liquid–solid ratio, X_2_ is the first extraction time, and X_3_ is the second extraction time.

**Figure 3:**
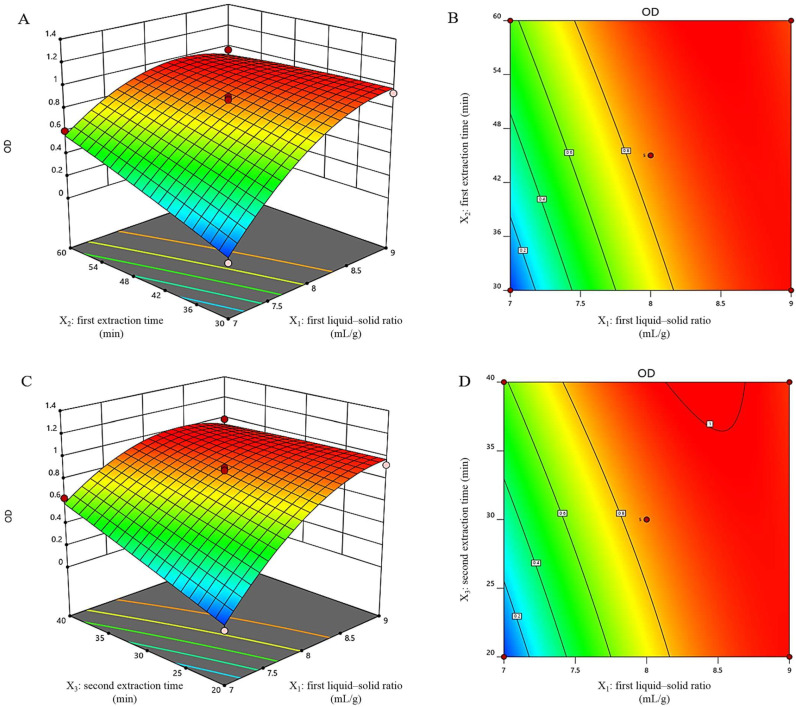
The interaction surface (**A**) and the contour diagram (**B**) of the first liquid–solid ratio (X_1_) and the first extraction time (X_2_); the interaction surface (**C**) and the contour diagram (**D**) of the first liquid–solid ratio (X_1_) and the second extraction time (X_3_).

A further ANOVA was performed on the regression model (Table S3). The table showed that the model was statistically significant (*p* < 0.05) and that the linear terms (X_1_, X_2_, and X_3_) had a significant (*p* < 0.05) effect on extraction. The interaction surfaces and contour plots of X_1_, X_2_, and X_3_ are shown in Figure 3A–D. The plots showed that when the first extraction time (X_2_) or the second extraction time (X_3_) was fixed at a certain level, the OD value increased rapidly alongside the increase in the first liquid–solid ratio (X_1_) and then decreased slightly. This indicated that the increase in the first liquid–solid ratio was not always conducive to the response and that saturation may have occurred. By adjusting the liquid–solid ratio appropriately, two compounds could be yielded more efficiently. The interaction surfaces and contour plots between X_2_ and X_3_ are shown in Figure S2. In cases where the second extraction time was fixed at a certain level, the OD score increased with the first extraction time; however, this increase was small. Perhaps the increased extraction time resulted in a more efficient destruction of the plant cells, resulting in a higher extraction rate; however, this interaction was not the primary cause. 

The optimal extraction conditions for YQD were calculated by solving the model’s extreme values and analyzing the contours. The first liquid–solid ratio was 8.086, the first extraction time was 59.998 min, and the second extraction time was 39.998 min. The optimal extraction process resulted in an OD value of 1.06. Due to actual manufacturing considerations, the optimum conditions of YQD were as follows: a liquid–solid ratio of 8, the first extraction time was 60 min, and the second extraction time was 40 min.

#### 2.2.2. Experimental Validation of the Model

The optimized YQD extraction process was as follows: 100 g of the prescription amounts of the HQ, YZZ, CWZ, and JQG pieces were blended together and extracted with eight times the amount of water. After being soaked for 30 min, fire-boiled, and decocted on a small fire for 60 min, the mixture was immediately filtered through a 200-mesh screen. The filter residue was then extracted with seven times the amount of water, fire-boiled, decocted on a small fire for 40 min, and immediately filtered again. The twice-combined extraction decoction was concentrated to 0.2 g/mL under reducing pressure at 60 °C.

Six validation experiments were conducted to verify the above optimal solution. The OD value was 0.96 and the average contents of calycosin-7-*O*-β-d-glucoside and chlorogenic acid were 0.107 mg/g and 0.338 mg/g, respectively. Table S4 contains the detailed extraction rates.

### 2.3. Identification of the Constituents of YQD and Optimization of the Quality Control Methods

#### 2.3.1. Identification of Chemical Constituents 

**Figure 4:**
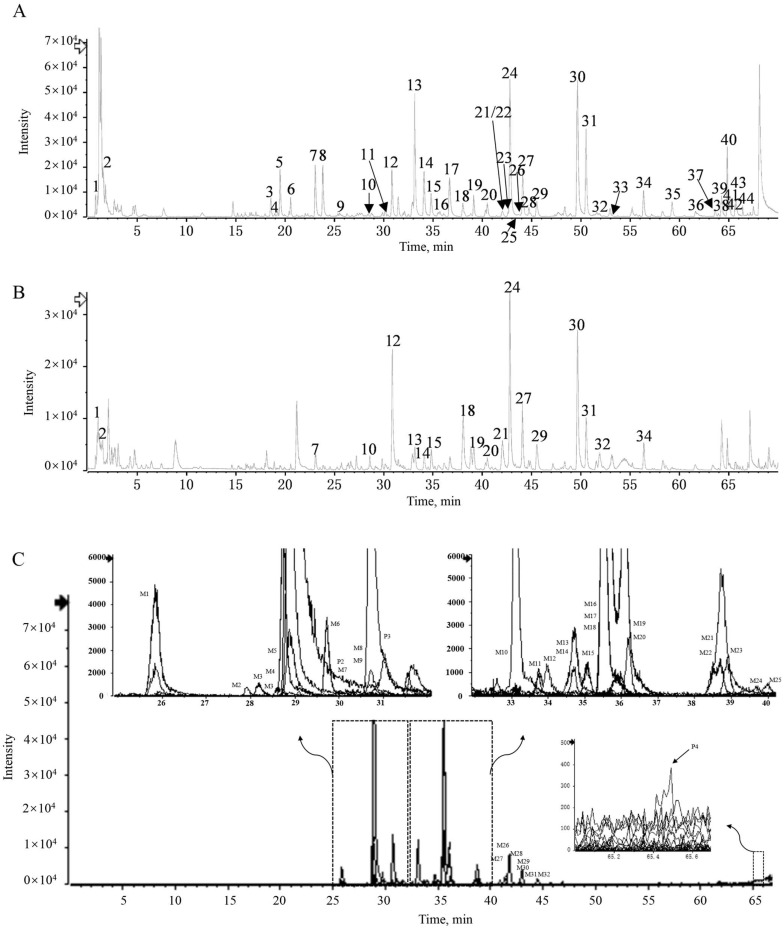
(**A**) Base peak chromatogram (BPC) of the YQD sample in negative ion mode; (**B**) base peak chromatogram (BPC) of the YQD sample in positive ion mode; and (**C**) extracted ion chromatograms (EIC) of absorbed components and metabolites after oral administration of YQD at 1 h.

UPLC-Q-TOF/MS was used for qualitative analysis of YQD. First, the MS data were matched with the Natural Products HR-MS/MS Spectral Library 1.0 database. Compounds not included in the database were identified in the literature and mass spectrometry pyrolysis laws. Finally, 44 chemical compositions (C1–C44) were identified in YQD (Figure 4A,B and Table 3). Among them, C12 from HQ and C13, C24, C30, and C31 from YZZ had higher response values. Typical compounds of flavonoids and triterpenoid saponins were used to analyze the cleavage pattern as follows:

C12 (C_22_H_22_O_10_, 30.86 min) showed a quasi-molecular ion at *m*/*z* 447.1269. The neutral loss of glucose (C_6_H_10_O_5_, −162 Da) in the MS^2^ spectrum formed a fragment ion *m*/*z* 285.0750, which further lost a methyl (•CH_3_, −15 Da), and the methoxy group (CO, −28 Da) formed the fragment ions *m*/*z* 270.0517 and *m*/*z* 242.0541. Fragment ion *m*/*z* 285.0750 may also lose methanol (CH_3_OH, −32 Da) and further lose the methoxy group (CO, −28 Da) to obtain the fragment ion *m*/*z* 225.0522. Fragment *m*/*z* 137.0217 may be obtained via the RDA reaction of the compound or the deglycosylated glycoside element. C12 was identified as calycosin-7-*O*-β-d-glucoside by comparing its MS^2^ cleavage behavior and retention time with those of the reference compounds. 

**Figure 5:**
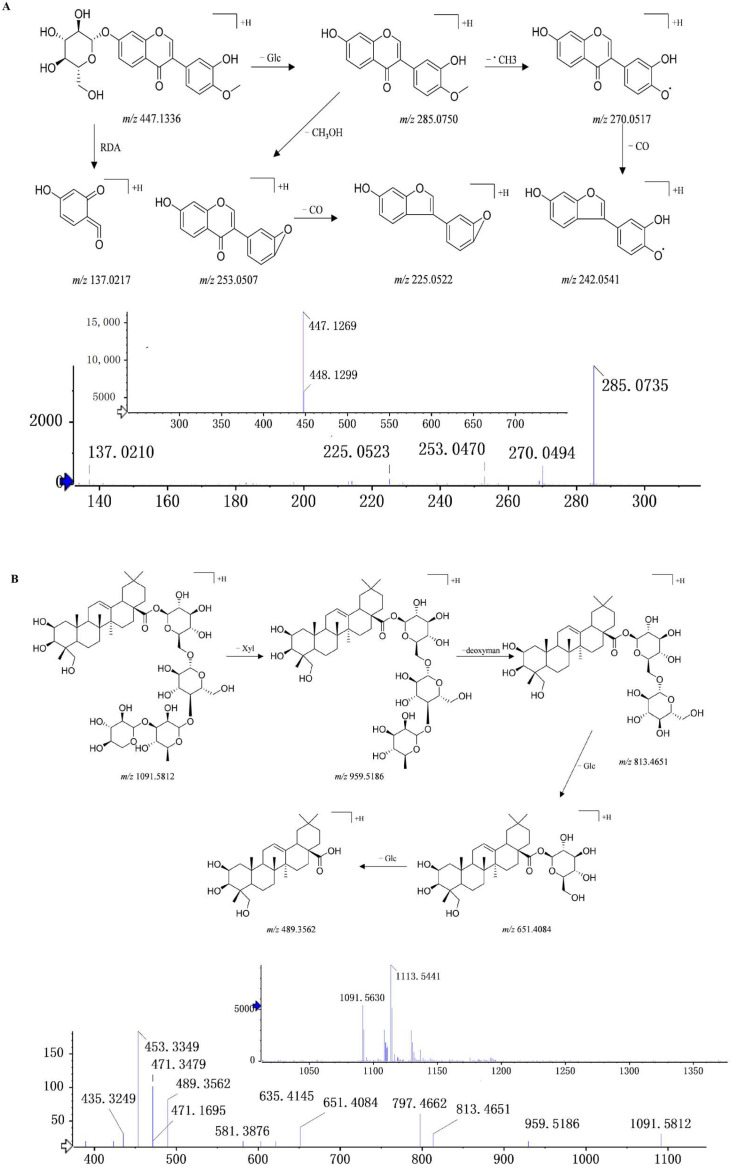
MS^2^ and the fragmentation pathways of (**A**) calycosin-7-*O*-β-d-glucoside and (**B**) abekia saponin.

C24 (eluted at 42.79 min) showed a quasi-molecular ion at *m*/*z* 1091.5812 [M + H]^+^, with a molecular formula of C_53_H_86_O_23_ and fragment ions at *m*/*z* 959.5186, 813.4651, 651.4084, and 489.3562, which were generated by the quasi-molecular ion peaks losing one molecule of xylose, one molecule of deoxymannose, and one molecule of glucose in turn; the fragment ions at *m*/*z* 489.3562 was arjunolic acid sapogenin. Using data from reference [31,32], C24 was identified as abekia saponin. The MS^2^ spectrum and fragmentation pathway of C12 and C24 are shown in Figure 5. All of the above results are beneficial for the further discovery of the absorbed ingredients after oral administration.

#### 2.3.2. Identification of Absorbed Components in Rat Plasma 

A total of 36 components of YQD were identified from rat plasma using background reduction, baseline correction, and molecular characterization extraction methods, of which, 4 were prototype components (P1–P4) and 32 were metabolites (M1–M32). Results are shown in Figure 4C and Table 4. The prototype components were stachydrine (P1), carasinaurone (P2), calycosin-7-*O*-β-d-glucoside (P3), and akebia saponin B (P4). Among them, P1 and P3 are the quality control indicators of HQ and CWZ, respectively, in the *Chinese Pharmacopoeia* (2022) [33]. P2 and P4 are the signature active ingredients of JQG and YZZ, respectively [34,35]. Due to low oral bioavailability and a short half-life, the components with high responses in vitro (C13, C24, C30, and C31) were not found as prototypes or metabolites in vivo [36,37,38]. By studying the metabolic pathways in detail, we found that the metabolites were mainly from glucuronidation, sulfation, hydroxylation, and demethylation reactions of the flavonoids in YQD. 

Glucuronidation: M2 showed a quasi-molecular ion at *m*/*z* 621.1487 in the negative ion mode that was 176 Da higher than calycosin-7-*O*-β-d-glucoside at *m*/*z* 447.1286, suggesting that calycosin-7-*O*-β-d-glucoside had undergone a glucuronidation reaction. The fragment ions of M2 at *m*/*z* 283.0620 and 268.0344 were like calycosin-7-*O*-β-d-glucoside; therefore, it was speculated that M2 was a glucuronidation of calycosin-7-*O*-β-d-glucoside. Similarly, the metabolites M10, M23, M6, and M27 were speculated to be glucuronide products of apigenin, formononetin, genkwanin, and methylnissolin, respectively. 

Sulfation: M32 showed a quasi-molecular ion at *m*/*z* 347.0230 in the negative ion mode that was 80 Da higher than formononetin at *m*/*z* 267.0663, indicating that a sulfation reaction had occurred in formononetin. Fragment ions of M32 at *m*/*z* 267.0650 and 252.0431 were similar to formononetin; therefore, it was speculated that M32 was a sulfated metabolite of formononetin. 

Glucuronidation + Sulfation: in the negative ion mode, M5 showed a quasi-molecular ion at *m*/*z* 525.0359, a fragment ion at *m*/*z* 269.0465, and was speculated to be the sulfated and glucuronidated product of apigenin because the quasi-molecular ion was 176 and 80 Da higher than that of apigenin at *m*/*z* 269.0455, and the fragment ion was similar to apigenin.

Demethylation: M24 was tentatively identified as a demethylated product by comparing the quasi-molecular ion between M24 and formononetin; the gap was 14 Da in negative ion mode. 

**Figure 6:**
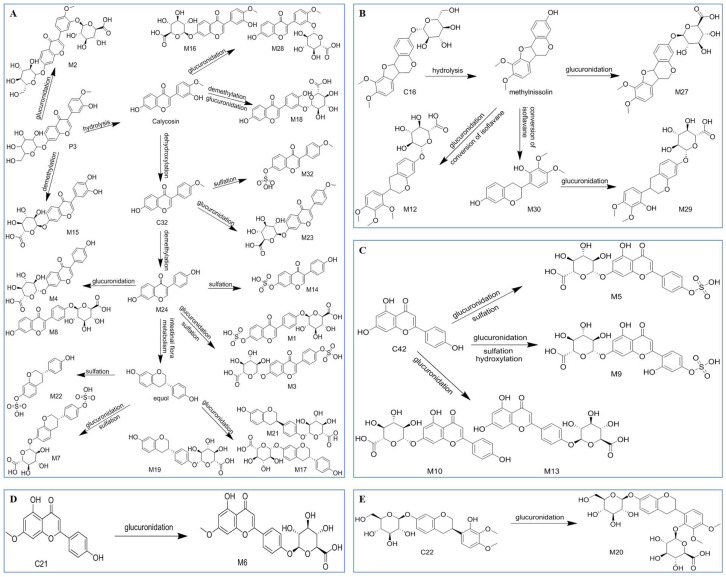
Possible metabolic pathways of (**A**) calycosin-7-*O*-β-d-glucoside; (**B**) methylnissolin-3-*O*-glucoside; (**C**) apigenin; (**D**) genkwanin; and (**E**) astraisoflavan-7-*O*-β-d-glucoside.

In addition, M30 showed a quasi-molecular ion at 303.1212 and fragment ions at *m*/*z* 303.1024, 167.0699, 133.0677, and 123.0434 and so was hypothesized to be isoflavan transformed by methylnissolin [39]. The [M − H]^−^ *m*/*z* of M9 was 541.0301, which was 176, 80, and 17 Da higher than that of apigenin. It was speculated that M9 was the hydroxylation, sulfation, and glucuronidation product of apigenin. The possible metabolic pathways are shown in Figure 6.

#### 2.3.3. Establishment of the SPE-UPLC-Q-TOF/MS Identification Method

Identification is an important part of the quality standard of TCM. Traditional TLC or HPLC methods often have low sensitivity, many interfering factors, and complicated pretreatment methods that cannot meet the identification needs of TCM compound preparations. In this study, the SPE-UPLC-Q-TOF/MS technique was used to identify the characteristic peaks of YQD; the separation of the components was better after SPE extraction. Asperulosidic acid (C3) and carasinaurone (C11) in the H_2_O eluting phase; neochlorogenic acid (C5), chlorogenic acid (C7), and cryptochlorogenic acid (C8) in the 30% methanol elution phase; and calycosin-7-*O*-β-d-glucoside (C12) and calceolarioside B (C13) in the 60% methanol elution phase were selected as the characteristic components because they were from four separate herbs with high response values, complete separation, and good peak shapes. 

**Figure 7:**
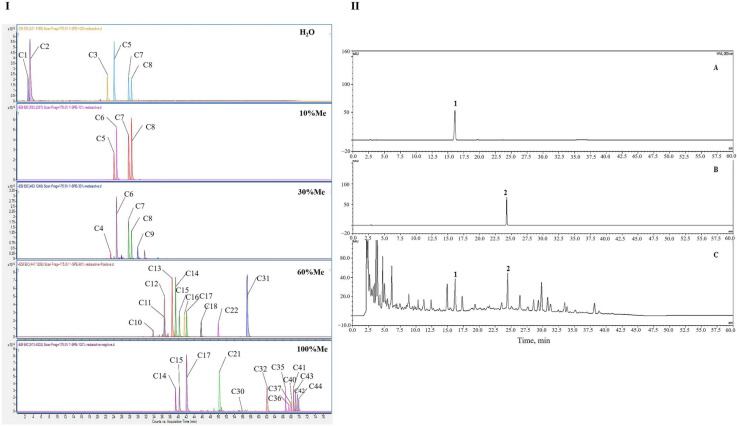
(**I**): EIC diagram of YQD detected after SPE segmentation. (**II**): HPLC chromatograms of reference (**A**,**B**) and the YQD sample (**C**). (1. chlorogenic acid; 2. calycosin-7-*O*-β-d-glucoside).

The results of sample identification showed that, in the H_2_O elution section, the retention times of C3 and C11 were 21.81 and 36.12 min, respectively; in the 30% methanol elution section, the retention times of C5, C7, and C8 were 23.46, 27.17, and 27.91 min, respectively; and in the 60% methanol elution section, the retention times of C1 and C13 were 36.41 and 38.38 min, respectively (Figure 7I).

#### 2.3.4. Establishment of HPLC Quantitative Method

An HPLC method was established for the determination of calycosin-7-*O*-β-d-glucoside (CAG, C12) and chlorogenic acid (CA, C7). CAG is a representative component of HQ [33]. CA may promote melanogenesis in melanoma cells [40] and had a higher response value in the UPLC/MS chromatogram. Therefore, these two substances were selected as quantitative components to compensate for the lack of quantitative analysis in the existing quality standards.

As can be seen from Figure 7II, the response value of the target peak was high, the separation degree was good, and the other peaks did not interfere with the measurement. The standard curves were Y = 0.292X–0.3234, *r* = 0.9998 (CAG) and Y = 0.099X–0.0567, *r* = 1.000 (CA). The linear ranges for CAG and CA were 5.21–208.4 μg/mL and 10.32–412.8 μg/mL, respectively. The CAG and CA control solutions were taken separately, and the peak areas were measured via successive injections six times according to the established HPLC conditions. The results showed that the RSDs of CAG and CA were 0.25% and 1.63%, respectively (*n* = 6), which indicated that the precision of the instrument was good. For the evaluation of repeatability, the RSDs of the peak areas from six samples of the same batch were calculated and were 0.77% and 3.31%, respectively (*n* = 6), indicating that the repeatability of the method was good. The stability of the samples was determined by re-analyzing the RSDs of the peak area from one prepared sample at 0, 2, 4, 6, 8, 12, and 24 h. The RSDs of CAG and CA were 0.54% and 3.97%, showing that the sample solutions were stable within 24 h at room temperature. The recoveries were calculated using three concentrations (equivalent to 0.5, 1.0, and 1.5 times the original amounts in the samples), and the recovery rate of CAG was 92.25–98.63% and RSD was 0.87–2.30%; the recovery rate of CA was 97.78–105.30% and the RSD was 2.97–3.46%, which indicated that the method had a high recovery rate and good accuracy. 

Using the newly established method, ten batches of YQD samples were evaluated. The results (Table S5) show that the content of CAG and CA were 0.333–0.484 mg/g and 0.275–0.383 mg/g, respectively. The RSD was less than 15%, indicating that the method could conduct the quantitative analysis of the two components. Under the analytical conditions, the response value and resolution of CAG and CA met the quantitative requirements well.

### 2.4. Network Pharmacology Analysis and Molecular Docking Studies 

#### 2.4.1. Bioactive Ingredients and Potential Targets of YQD 

Network pharmacology emphasizes the multi-level and multi-angle discovery of potential targets and signaling pathways. This includes identifying active ingredients, their targets, disease targets, and screening the core genes and then constructing networks within the compound–target–disease–signaling pathway and evaluating the effects of compounds on these networks [41,42]. The ingredients comprising TCM preparations are numerous; however, the components present at the highest amounts or those that can be readily absorbed by the body may be the active components. Therefore, the compounds found in rat plasma were subjected to further network pharmacology analysis. As the metabolites of daudzein and equol (M1, M3, M4, M7, M8, M14, M17, M19, M21, and M22) are derived from diverse sources [43], they were not specific metabolic components of YQD and were therefore excluded. Additionally, apigenin (C42), a prototype component that produces many metabolites, and four components (C13, C24, C30, and C31) with high response values in the UPLC/MS chromatogram were added to the pharmacological prediction model. The 31 compounds used for network pharmacology analysis are shown in Table 5. In total, 466 targets related to the compounds were found (Table S6), including AKT1, TNF, ALB, mTOR, MAPK1, IL2, PIK3CA, IGF1, and others.

#### 2.4.2. PPI and CTD Network Analysis 

**Figure 8:**
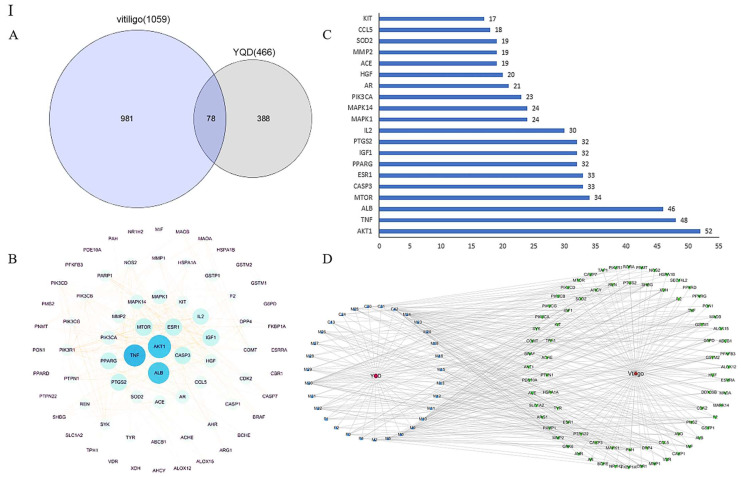

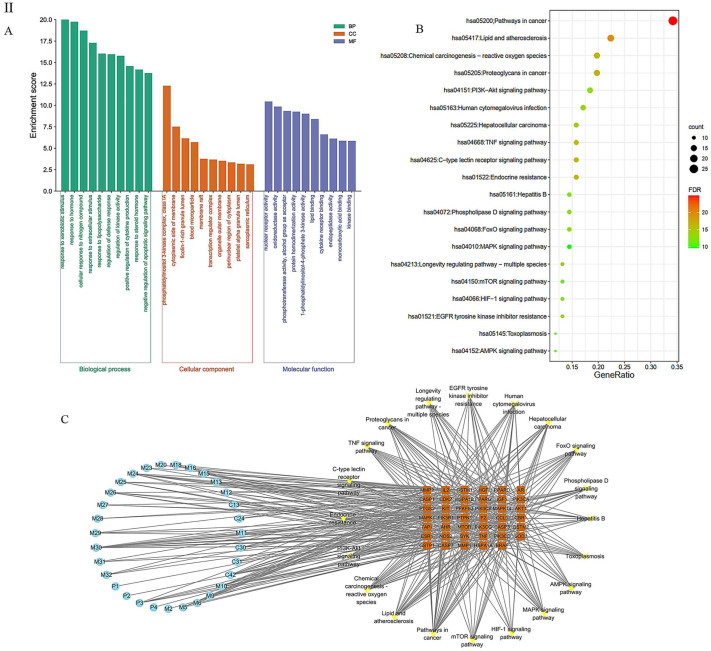
**I**: (**A**) Venn diagram of targets related to YQD and vitiligo. (**B**) Visualization analysis of the PPI diagram—the size of the circle represents the target degree. (**C**) Adjacent nodes of the overlapping targets between YQD and vitiligo—the X-axis represents the number of adjacent proteins of the target and the Y-axis represents different targets. (**D**) Compound–target–disease (CTD) network—31 components (blue) were connected to 78 targets (green) and YQD (red), and 78 target genes were connected to 31 components and vitiligo (red). **II**: (**A**) GO analysis. (**B**) Top 20 enriched KEGG pathways. (**C**) Compound–target–pathway (CTP) network—31 components (blue) were connected to 41 targets (orange), and the 41 targets were connected to 20 pathways (yellow) and 31 components.

The target genes related to vitiligo were searched, and 78 overlapping targets were identified by matching the 466 YQD targets with the 1059 vitiligo genes (Figure 8I(A), Table S7). Based on these 78 targets, a PPI network was successfully constructed and visualized (Figure 8I(B), Table S8). The top 20 targets with degree values were selected as potential core targets (Figure 8I(C)) and included AKT1, mTOR, and MAPK1. Phosphorylated AKT1 has been reported to increase the transcription of MITF, a major regulator of melanin synthesis, which further upregulates the expression of TYR, TRP-1, and TRP-2 and ultimately stimulates melanin synthesis [44]. mTOR activation was beneficial for melanocyte protection from dendritic loss under oxidative stress, thereby maintaining melanin levels in skin pigmentation [45]. Early expression of phosphorylated MAPK1 can upregulate the transcriptional activity of MITF, thereby promoting melanin synthesis [46].

To investigate the underlying relationship mechanism of YQD on the therapy of vitiligo, a compound–target–disease (CTD) network was constructed, as shown in Figure 8I(D). There were 111 nodes in the network, with 31 components, 78 targets, 1 illness (vitiligo), and 1 drug (YQD). The average degree value of the compounds was 6.79, with 16 compounds having a degree value greater than 6 (Table S9). Among them, M30, M24, C42, M32, M10, M13, M15, M6, P3, and C31 ranked in the top ten; the seven metabolites were derived from C42, C16, C32, C12, and C21, respectively. Research has shown that apigenin (C42) may target TRP-1 and TRP-2 through the MAPK pathway, thereby promoting melanin production [47]. Akebia saponin D (C31), formononetin (C32), and genkwanin (C21) have demonstrated good anti-inflammatory, antioxidant, and immunomodulatory activities [48,49,50,51]. 

#### 2.4.3. GO and KEGG Enrichment Analysis 

According to GO classification enrichment analysis, the vitiligo targets were categorized as being involved in the stress response, activation of cytokines, biological regulation, and cell communication (Figure 8II(A)). The top 20 vitiligo-related pathways included the TNF, FoxO, PI3K-Akt, and mTOR signaling pathways according to KEGG enrichment (Figure 8II(B) and Table S10). 

To validate that the ingredients could regulate the targets through the aforementioned pathways to produce the therapeutic effects, we constructed a compound–target–pathway (CTP) network using 41 core target genes (in the top 20 pathways) (Figure 8II(C)). The network had 92 nodes, including 31 components, 20 pathways, and 41 targets. M30, C42, M10, M15, M24, M13, M6, and P3 had higher than average degree values in the network, whereas the targets with higher degree values were AKT1, MAPK1, PIK3CA, and mTOR. The results were consistent with the PPI and CTD network analyses. The PI3K-Akt, TNF, MAPK, and FoxO signaling pathways were enriched for more than 10 targets (Table S11) that have been reported in the literature as potentially critical pathways for vitiligo treatment [52,53,54]. 

The results of network pharmacology showed that YQD is a multi-component, multi-target, and multi-pathway treatment for vitiligo. Calycosin-7-*O*-β-d-glucoside (C12), methylnissolin-3-*O*-glucoside (C16), genkwanin (C21), akebia saponin D (C31), formononetin (C32), and apigenin (C42) may be the key components that regulate multiple targets to perform therapeutic roles. 

#### 2.4.4. Molecular Docking Study 

Molecular docking is an alternative technique for predicting the binding affinity of molecules to the active site of a particular receptor without the use of animals; it enables high-throughput screening of components [55,56]. It is generally accepted that the lower the energy when the conformation of the ligand binding to the receptor is stable, the great-er the probability of action. Target prediction in network pharmacology is heavily reliant on the predictive capacity of network prediction tools, and the data are primarily generated from experiments [57]. However, because of the restricted number of experimentally confirmed drug targets, certain less-studied TCMs projected fewer targets, causing their constituents to be excluded from the active ingredients.

**Figure 9:**
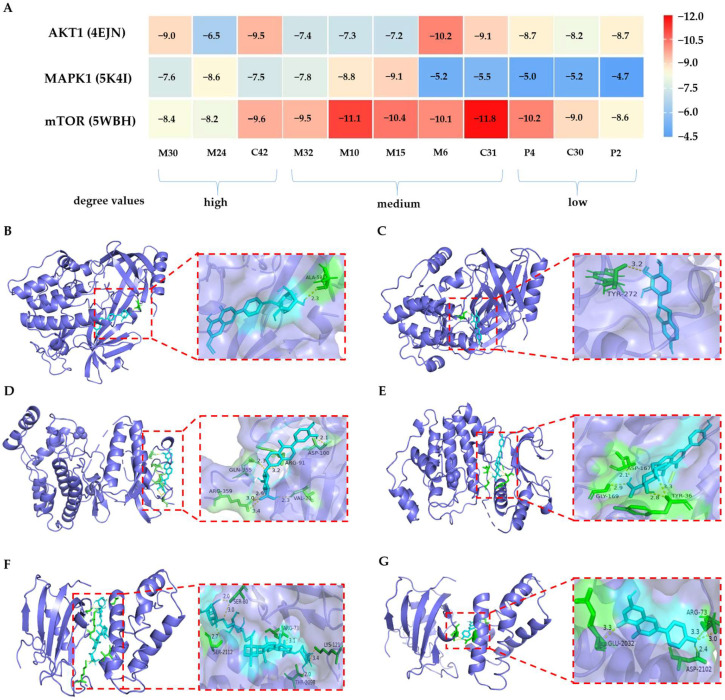
(**A**) Heatmap of the predicted molecular docking of the predicted active compounds with three core targets; (**B**) M6 and AKT1; (**C**) M30 and AKT1; (**D**) M15 and MAPK1; (**E**) M10 and MAPK1; (**F**) C31 and mTOR; and (**G**) P4 and mTOR.

In this study, the predicted active compounds with high (M30, M24, and C42), medium (M32, M10, M15, M6, and C31), and low (P4, C30, and P2) degree values in network pharmacology were docked with potential core targets (AKT1, MAPK1, and mTOR) to verify the predicted results and explore the possibility of compounds with a low degree value participating in the binding of targets. As shown in Figure 9A, the binding energies of the 11 ligands to the core targets ranged between −4.7 and −11.8 kcal/mol, which implies that all of them had good binding activities to the proteins except for P2/MAPK1 (−4.7 kcal/mol). We found that the strongest binding ability to AKT1 was with M6 and M30, which formed hydrogen bonds with residues ALA-58 (Figure 9B) and TYR-272 (Figure 9C) that had binding energies of −10.2 and −9.0 kcal/mol, respectively. The component with the strongest binding capacity to MAPK1 was M15, and the hydrogen bond binding sites were at ARG-359, VAL-21, GLN-355, ARG-91, and ASP-100 (Figure 9D). M10/MAPK1 ranked second and formed hydrogen bonds with residues ASP-167, GLY-169, and TYR-36 (Figure 9E). The binding energy of the 11 ligands to mTOR ranged between −8.2 and −11.8 kcal/mol, with C31/mTOR having the lowest binding energy and forming hydrogen bonds with five residues (Figure 9F). In addition, P4 is predicted to bind strongly to mTOR, forming hydrogen bonds with GLU-2032, ASP-2102, and ARG-73 (Figure 9G). The results suggest that the components with lower predicted activity determined using network pharmacology may also have a strong binding capacity to core proteins and thus exert therapeutic effects.

In conclusion, combining the above computational results and considering the metabolic pathways of the metabolites, we hypothesized that C11 (carasinaurone, JQG), C12 (calycosin-7-*O*-β-d-glucoside, HQ), C16 (methylnissolin-3-*O*-glucoside, HQ), C21 (genkwanin, CWZ), C31 (akebia saponin D, YZZ), C32 (formononetin, HQ), C40 (akebia saponin B, YZZ), and C42 (apigenin, HQ) may be the pharmacodynamic markers of YQD that deserve further in-depth study.

### 2.5. Effects of YQD on Melanogenesis In Vitro and In Vivo 

#### 2.5.1. Effects of YQD on Melanogenesis in B16F10 Cells

**Figure 10:**
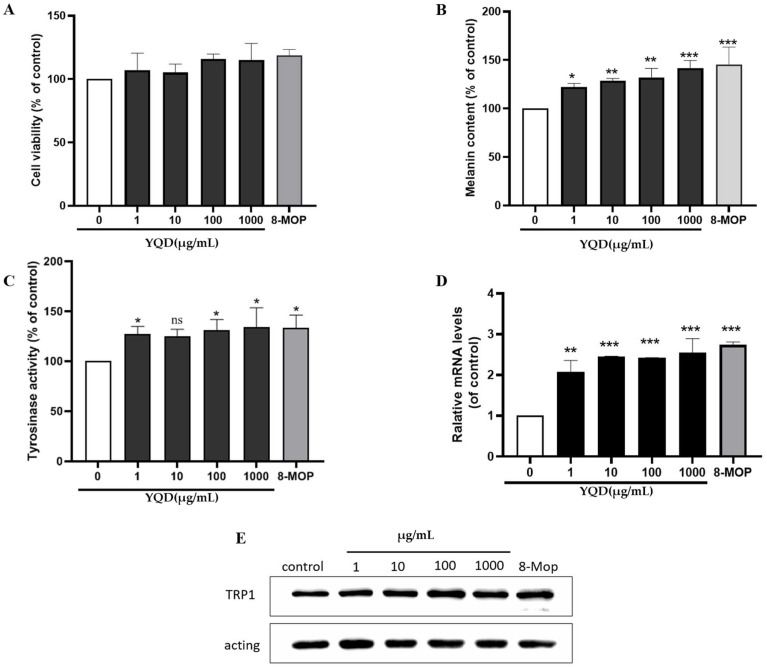
YQD promotes melanogenesis in B16F10 cells. The B16F10 cells were treated with YQD at different concentrations (1–1000 µg/mL), medium alone (control), or 8-MOP (positive control, 100 μmol/L) for 48 h: (**A**) effect on cell viability examined using the CCK-8 assay; (**B**) melanin content of cells measured by NaOH assay; (**C**) tyrosinase activity measured by tyrosinase activity assay; (**D**) expression of the TRP-1 gene analyzed via qRT-PCR; and (**E**) levels of TRP-1 proteins evaluated using Western blotting. (ns: no significance, * *p* < 0.05, ** *p* < 0.01, and *** *p* < 0.001).

The viability of the murine B16F10 melanoma cell line was examined in the presence of different concentrations of YQD using the CCK-8 assay. The results showed that B16F10 cells treated with YQD at concentrations of 1–1000 µg/mL for 48 h did not display any changes in cell viability when compared with the control (Figure 10A). 

To determine the effects of YQD on pigmentation, we examined the melanin content of B16F10 cells treated with different concentrations (1–1000 µg/mL) of YQD and a positive control drug (8-MOP) for 48 h. As shown in Figure 10B, YQD increased the melanin content in B16F10 cells in a dose-dependent manner.

To investigate its effect on the melanogenic pathway in B16F10 cells, we explored whether YQD affected TYR activity and the expression of the melanin biosynthetic gene (TRP-1). As shown in Figure 10C, the activity of cellular TYR increased significantly in a concentration-dependent manner following incubation with YQD. The results of the qRT-PCR experiments showed that the mRNA level of TRP-1 was elevated after YQD treatment (Figure 10D); moreover, Western blotting showed that the protein level was also increased significantly in a dose-dependent manner (Figure 10E). These results indicate that YQD induced pigmentation by enhancing TYR activity and upregulating TYR family genes.

#### 2.5.2. Effects of YQD on Extracellular TYR Activity

An in vitro TYR activation assay showed that YQD could activate mushroom TYR and that the activation rate was concentration dependent (Table S12). The TYR activation rate for 8 mg/mL of YQD reached 79.66%, which was equivalent to the activation rate for 0.5 mg/mL of 8-MOP.

#### 2.5.3. Effects of YQD on Melanogenesis in Zebrafish

After observing the effects of YQD in mammalian cells, we wanted to determine whether a model organism had a similar response. Zebrafish embryos were used to investigate the role of YQD in promoting melanogenesis in vivo. Firstly, the acute toxicity and dose were examined by observing pericardial edema, delayed yolk absorption, and mortality in zebrafish embryos within 96 h postfertilization (hpf) in the presence of YQD. The statistical results are shown in Table S13, and the condition of zebrafish 72 hpf in the presence of YQD is depicted in Figure S3A. The results show that concentrations of YQD below 1000 μg/mL had no influence on zebrafish embryos. However, a considerable number of juvenile fish died in both groups at 2000 μg/mL (the mortality rates were 50% and 100%). All embryos had died 72 hpf when the concentration was increased to 4000 μg/mL. The calculated LC_50_ was 1997 μg/mL (Figure S3B). 

**Figure 11:**
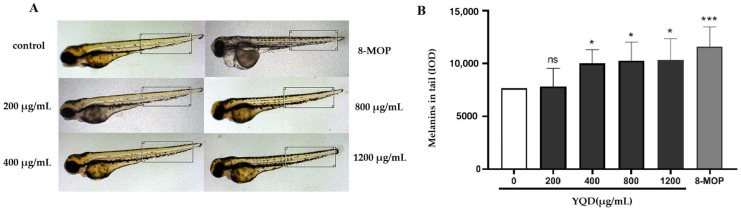
YQD promotes melanogenesis in zebrafish. The zebrafish embryos were exposed to E3 buffer (control), varying doses of YQD (200–1200 μg/mL), or 8-MOP (positive control, 100 μmol/L) from 24 hpf (hours postfertilization) to 84 hpf. The melanin densities (IOD) in the tails were measured using Image-Pro Plus: (**A**) melanin in the tails of zebrafish 84 hpf; (**B**) melanin densities in zebrafish. (ns: no significance, * *p* < 0.05, *** *p* < 0.001).

Next, we explored the effects of YQD on melanogenesis. Zebrafish embryos were separated into eight groups and constantly treated with blank culture medium, varying doses of YQD (200–1200 μg/mL), or a positive control drug (8-MOP) from 24 hpf to 84 hpf. The density and distribution of the melanin granules were observed and recorded every day. We found that the melanin density in the head and tail gradually increased as the embryos grew. The difference between groups peaked at 84 hpf; melanin production in the head, dorsal spine, and abdomen of zebrafish treated with YQD was more noticeable than in other parts of the body, whereas the survival and development of juvenile fish were not affected in the high-concentration treatment group (Figure 11A). We measured the melanin density in the tails of zebrafish and found that the melanin density in the YQD-treated groups (400–1200 μg/mL) was significantly higher than that in the control group (Figure 11B).

## 3. Materials and Methods 

### 3.1. Materials and Regents 

HQ, YZZ, CWZ, and JQG were collected from different parts of China. The origins and batch numbers are shown in Table S14. Methanol and acetonitrile (both liquid chromatography grade) were purchased from Merck (Darmstadt, Germany) and formic acid was acquired from ANPEL Lab Tech. (Shanghai, China). 8-Methoxypsoralen (8-MOP) was purchased from Macklin Biochemical Co., Ltd. (Shanghai, China). The solid-phase extraction (SPE) cartridges (Oasis HLB, 3 mL) were purchased from Waters (Milford, MA, USA). Pure water (18.2 MΩ/cm) was deionized using a Milli-Q system (Millipore, Bedford, MA, USA).

### 3.2. Statistical Analysis of the HPLC Fingerprints

Chromatographic data from 15 different batches of each herb were mined and analyzed. Similarity analysis was performed using the “Chinese Materia Medica chromatographic fingerprint similarity evaluation system” (ver. 2012) [20]. The time window width was set to 0.1 min, and the median method was selected for full-spectrum peak matching. The simulated average fingerprint R was generated, the similarity between the sample and the average fingerprint R was calculated, and then the common peaks that existed in each herb were observed [21]. A chemometric analysis including HCA and PCA was used to examine the similarities and differences between the samples [22]. SIMCA software (ver. 14.1) was used to categorize the samples, and dendrograms were drawn to characterize the classification results. Significant PCs were extracted under the condition that the corresponding eigenvalues were greater than 0.99. A score plot was used to assess sample variation and PC comprehensive scores were calculated to evaluate the sample quality.

### 3.3. Preparation of the HQ, YZZ, CWZ, and JQG Decoction 

An amount of 100 g of the HQ, YZZ, CWZ, or JQG pieces was extracted using 10 times the amount of water. After being soaked for 30 min and decocted for 60 min, the mixture was immediately filtered through a 200-mesh screen. The filtrate residue was then extracted with 8 times the amount of water, decocted for 40 min, and immediately filtered again. The twice-combined extraction decoction was concentrated to 0.2 g/mL through reducing pressure [19].

### 3.4. Optimization and Validation of the YQD Preparation Conditions

A three-factor with three levels (−1, 0, +1) BBD was applied to optimize the critical parameters, such as the first solid–liquid ratio, first extraction time, and second extraction time [28]. The three responses used to evaluate the influence of the extraction parameters were the calycosin-7-*O*-β-d-glucoside and chlorogenic acid contents of the extract and its total solid content, and the OD value was used to express the overall impact results. Multiple regression analysis was used to develop the best-fit polynomial quadratic model [22]. The *p*-value, lack of fit value, and coefficient of determination (*R*^2^) from the ANOVA were used to assess the model adequacy. Experimental design, statistics, response surfaces, and contour plots were obtained using Design-Expert software (ver. 12.0).

After optimizing the preparation conditions of YQD, six validation experiments were conducted for the optimal solution, with a first liquid–solid ratio of 8, a second liquid–solid ratio of 7, a first extraction time of 60 min, and a second extraction time of 40 min. The experimental values were compared with the predicted ones to validate the model.

### 3.5. Preparation of the Sample and Standard Solution 

HPLC and UPLC-TOF/MS samples: an amount of 1 mL of each decoction sample (0.2 g/mL) was accurately extracted, centrifuged at 13,000 rpm for 10 min, and then the supernatant was filtered through a 0.22 μm membrane before use. 

SPE-UPLC-TOF/MS samples: an amount of 500 μL of YQD was added to 500 μL of deionized water, mixed thoroughly, and centrifuged at 12,000 rpm for 15 min. Then, 1 mL of the supernatant was subjected to the activated SPE column and eluted successively with H_2_O, 30%, 60%, and 100% methanol (1 mL for each). The flow rate was controlled uniformly (about 10 drops/min). The eluent was collected and dried, redissolved in 100 μL of 50% methanol, vortexed for 3 min, sonicated for 3 min, and centrifuged at 12,000 rpm for 15 min, and the supernatant was then taken for analysis.

Standard solutions: stock solutions of calycosin-7-*O*-β-d-glucoside and chlorogenic acid were separately prepared in methanol. The stock solutions of each analyte were diluted in 60% methanol to prepare calibration solutions and filtered through 0.22 μm membranes before use.

### 3.6. Animals and Drug Administration 

Sprague–Dawley rats (180–200 g, 6–7 weeks old) were provided by Shanghai Silaike Experimental Animal Co., Ltd. (Shanghai, China, approval number: 2021-0009). The rats were kept in a room (24 ± 2 °C with 60% ± 5% humidity) with a 12 h light/dark cycle for one week and had free access to water and conventional laboratory food to acclimate. The rats were randomly assigned to the control group or YQD group (*n* = 6 each). The rats in the YQD group were orally administered with YQD (dissolved in purified water) once a day at a dose of 15 g/kg (decoction pieces weight/body weight) for 5 consecutive days. The same volume of saline solution was given to the rats in the control group. Blood was collected 1 h after the last dosing. 

All experiments were conducted in accordance with the Guiding Principles for the Care and Use of Laboratory Animals and were approved by the Institutional Animal Care and Use Committee (IACUC) of Shanghai Jiao Tong University (approval number: A2021145, approval date: 23 December 2021). All work was performed following the recommendation of the ARRIVE guidelines.

### 3.7. Plasma Collection and Preparation 

The blood samples were collected and centrifuged at 4000 rpm for 10 min at 4 °C. The plasma samples were then transferred to Eppendorf tubes and kept at −80 °C for further analysis. To precipitate protein, 200 μL of plasma was treated with 800 μL of methanol, vortex mixed for 1 min, and centrifuged at 12,000 rpm for 15 min at 4 °C. The supernatant was transferred to a new tube and evaporated to dryness. For further analysis, the residue was redissolved in 80 μL of 50% methanol and centrifuged at 12,000 rpm for 15 min.

### 3.8. UPLC-Q-TOF/MS and HPLC Analysis 

For qualitative analysis, the samples were analyzed on an Agilent 1290 Infinity UPLC system (Milford, MA, USA) equipped with a Waters UPLC column (ACQUITY HSS T3, 2.1 × 100 mm, 1.8 μm). The mobile phase consisted of solvent A (0.1% formic acid aqueous solution) and solvent B (0.1% formic acid acetonitrile), and the gradient elution program was set as follows: 0–10 min, 100% A; 10–27 min, 100–85% A; 27–62 min, 85%–60% A; 62–70 min, 60%–5% A; 70–75 min, 5% A; and 75.01–78 min, 100% A. The flow rate was 0.3 and the column temperature was 30 °C. The injection volume was set at 5 μL. 

Mass spectrometric analyses were performed using an AB 4600 Q-TOF-MS/MS system (AB SCIEX, Framingham, MA, USA) equipped with an ESI source. The parameters for the mass spectrometry were as follows: scan range, 50–1800 *m*/*z* (MS) and 50–1250 *m*/*z* (MS/MS); ion spray voltage floating, −4.5 kV (negative mode) and 5.0 kV (positive mode); declustering potential, 100 V; source temperature, 500 °C; nebulizer gas, 50 psi; auxiliary heater gas, 50 psi; curtain gas, 35 psi; and collision energy, 10 eV (MS) and ±40 eV (MS/MS). To obtain the fragment information, N_2_ was used as the collision gas.

Quantitative analysis was performed on an UltiMate 3000 (Thermo, Waltham, MA, USA) liquid chromatograph system using an YMC C18 column (250 mm × 4.6 mm, 5 μm). The mobile phase was composed of solvent A (0.1% aqueous formic acid) and solvent B (acetonitrile), with the gradient elution program: 0–15 min, 95–85% A; 15–20 min, 85–80% A; 20–40 min, 80–65% A; 40–45 min, 65–95% A; 45–60 min, 95% A. The column temperature was maintained at 25 °C and flow rate was set at 1 mL/min. An amount of 10 μL of the solutions was injected and the wavelength was set to 260 nm.

### 3.9. Network Pharmacological Analysis 

The components discovered via UPLC-Q-TOF/MS were used to establish the chemical information database of YQD for network pharmacology research. We searched for the component-related genes with a possibility > 0.1 or z-score > 1.5 using the SwissTargetPrediction (https://www.swisstargetprediction.ch) and PharmMapper (https://www.lilab-ecust.cn/pharmmapper (both accessed on 2 April 2022)) online tools. Vitiligo-related genes were prepared from the GeneCards (https://www.genecards.org (accessed on 2 April 2022)), OMIM (https://www.omim.org (accessed on 24 March 2022)), DisGeNET (https://www.disgenet.org (accessed on 2 April 2022)), TTD (https://www.bidd.nus.edu.sg/group/cjttd (accessed on 24 March 2022)), and DrugBank (https://www.drugbank.ca (accessed on 24 March 2022)) databases. These target genes were standardized using the Uniprot database (https://www.uniprot.org (accessed on 2 April 2022)). Then, component-related targets and vitiligo-related targets were crossed using Venny (ver. 2.1.0) to obtain the potential targets of YQD in vitiligo treatment.

The protein–protein interaction (PPI) network was identified using the String Database (https://www.string-db.org (accessed on 28 May 2022)) and then imported into and visualized using Cytoscape (ver. 3.7.2). The Gene Ontology (GO) function and Kyoto Encyclopedia of Genes and Genomes (KEGG) pathway enrichment were performed via the Metascape database (https://metascape.org (accessed on 28 May 2022)). The compound–target–disease (CTD) and component–target–pathway (CTP) networks were constructed and analyzed using Cytoscape software (ver. 3.7.2).

### 3.10. Molecular Docking

The 3D structures of the ingredients and the crystal structures of targets were obtained from the Pubchem (https://www.ncbi.nlm.nih.gov) and PDB (https://www.rcsb.org (both accessed on 30 May 2022)) databases, respectively. The Autodock Tool (ver. 1.5.6) was employed for ingredient–protein molecular docking and calculating the binding affinity. The docking results were visualized using PyMOL (ver. 2.5.2). 

### 3.11. In Vitro Cells Assay

#### 3.11.1. Cell Cultures and Viability Assay

Mouse B16F10 melanoma cells (Chinese Academy of Sciences, Shanghai, China) were cultured in RPMI 1640 (Gibco, MA, USA) medium, supplemented with 10% FBS and a penicillin–streptomycin antibiotic mix in 5% CO_2_ at 37 °C.

The cell viability was measured using CCK-8 solution (Macklin, Shanghai, China) [24]. Briefly, B16F10 cells were seeded in 96-well plates (1 × 10^4^ cells/well) for 24 h. The cells were then treated with 8-MOP (100 μmol/L) as a positive control and different concentrations of YQD for 48 h. Then, the culture medium was removed and 10 μL of CCK-8 solution was added to each well. After incubation for 1 h at 37 °C, the absorbance was measured at 450 nm using a Varioskan Flash spectrometer (Thermo Scientific, Waltham, MA, USA). All the assays were performed in triplicate and the cell viability was calculated as (A_sample_/A_control_) × 100%.

#### 3.11.2. Melanin Measurement and TYR Activity Assay

Melanin release from B16F10 cells was measured using a previously described method with slight modifications [24]. B16F10 cells were seeded at a density of 1 × 10^4^ cells/well in a 6-well plate overnight. Then, the cells were treated with different concentrations of YQD and 8-MOP (100 μmol/L) for 48 h. Following the treatment, the cells were washed with ice-cold PBS and dissolved in 200 μL of 1 mol/L NaOH (with 10% DMSO) at 70 °C for 1 h. The absorbance at 490 nm was measured via a multiplate reader. The protein concentration of each sample was determined using the BCA Protein Assay Kit (Biomed, Beijing, China). The melanin content of the YQD-treated cells was calculated relative to the untreated cells. 

The assay for TYR activity was carried out as previously described [26], with a slight modification. B16F10 cells were incubated with test samples for 48 h and lysed with 1% Triton X-100 solution containing 1% sodium deoxycholate for 15 min at 0 °C. Each lysate was centrifuged at 12,000 rpm for 10 min to obtain the supernatant. After protein quantification and adjustment, 100 µL of the cell lysate was mixed with 100 µL of 0.1% L-DOPA (Yuanye Bio-Technology, Shanghai, China) and incubated at 37 °C for 30 min. The absorbance was measured at 475 nm using a multiplate reader, and the TYR activity in YQD-treated cells was presented relative to the activity in untreated cells.

#### 3.11.3. Quantitative Real-Time PCR 

Total cellular RNA was prepared from B16F10 cells treated with YQD using TRIzol reagent (solarbio, Beijing, China). cDNA was synthesized using Hifair III (Yeasen, Shanghai, China) according to the manufacturer’s instructions. qRT-PCR was performed at 95 °C for 5 min, followed by 40 cycles at 95 °C for 10 s and 60 °C for 30 s. The PCR primers used were as follows: 5′-GCCATCTTTGTCACCAGGTT-3′ (forward) and 5′-GCTCGAGCAAACTTCCATTC-3′ (reverse) for TRP1; 5′-CGCAGCCACTGTCGAGTC-3′ (forward) and 5′-GTCATCCATGGCGAACTGGT-3′ (reverse) for β-actin. The relative expression was determined by normalizing the data to the β-actin mRNA level.

#### 3.11.4. Western Blot Analysis

B16F10 cells were treated with different concentrations of YQD for 48 h. Cells were then lysed in cold RIPA buffer for 30 min on ice. The lysates were centrifuged at 12,000 rpm for 15 min at 4 °C and the supernatants were collected. The proteins were separated by 10% SDS-PAGE and then transferred onto PVDF membranes. The membranes were blocked with 5% skim milk, incubated with appropriate antibodies (TRP-1, ABclonal, Wuhan, China) at 4 °C overnight, and then incubated with the corresponding secondary antibodies for 2 h at room temperature. The targeted proteins were detected using the ECL detection system (GE Healthcare, Beijing, China) and visualized with the ChemiDoc XRS^+^ Imaging System (Bio-Rad, Hercules, CA, USA).

### 3.12. Extracellular TYR Activity

The Tyrosine Assay Kit (Yuanye Bio-Technology, Shanghai, China) was used to measure the extracellular TYR activity. L-DOPA, TYR, PBS, and different concentrations of YQD were added to the tubes according to the manufacturer’s instructions. The mixture was incubated for 10 min at 37 °C in a water bath; then, TYR was added and incubated for 2 min at 37 °C. The absorbance was determined at 475 nm and the activation rate was calculated. 

### 3.13. In Vivo Melanogenic Effect in Zebrafish 

Wild-type zebrafish embryos (AB strain) were collected from Shanghai Jiao Tong University. The embryos were incubated with E3 solution as a control and different concentrations of YQD from 24 h postfertilization (hpf) to 96 hpf at 28 °C. Two replications were set up for all groups (*n* = 30 in each group). The pericardium edema rate, yolk absorption delay rate, and mortality rate of zebrafish within 96 hpf were observed and recorded to investigate the toxicity of YQD. The LC_50_ was calculated using GraphPad Prism (ver. 8.0.2). 

In the melanin measurement assay [24], the embryos were incubated with 8-MOP (100 µmol/L) as positive control or variable concentrations of YQD from 24 hpf to 84 hpf. Three replications were set up for all groups (*n* = 10 in each group). A stereomicroscope (Olympus Instruments, Tokyo, Japan) was used to observe and record the melanin in the heads and tails of the zebrafish daily. The melanin density in the tail was measured using Image-Pro Plus (ver. 6.0). 

### 3.14. Statistical Analysis

All data were reported as the mean and standard deviation. The results were analyzed and illustrated using GraphPad Prism (ver. 8.0.2) software. The level of statistical significance is described in the respective figure legends (* *p* < 0.05, ** *p* < 0.01, and *** *p* < 0.001, as indicated by a one-way ANOVA).

## 4. Conclusions

In this study, a combination of fingerprints and chemometrics was used to select the optimal herbs for the preparation of YQD. The preparation process for YQD was optimized, and the chemical constituents and the absorbed constituents were identified. The SPE-UPLC-Q-TOF/MS identification method and HPLC quantification method were established to improve the quality standard of YQD. It was predicted that several signature components and metabolites from the four herbs might be important active components, reflecting the rationality of the YQ formula. In addition, B16F10 cell and zebrafish assays confirmed the role of YQD in promoting melanogenesis. The possible mechanism is that YQD can increase the expression and transcription of TRP-1 and enhance the activity of TYR, which is meaningful for the treatment of vitiligo. The results of this study fill many gaps in the research of YQ formulations and lay a good foundation for the clinical application and further development of YQD.

## Figures and Tables

**Table 1:** Rank of principal component scores and comprehensive scores for HQ, YZZ, CWZ, and JQG.

Sample (HQ/CWZ)	PC1 Scores	PC2 Scores	PC3 Scores	Comprehensive Scores	Rank	Sample (YZZ/JQG)	PC1 Scores	PC2 Scores	PC3 Scores	Comprehensive Scores	Rank
H-1	−0.96	0.34	−1.04	−0.58	12	Y-1	2.87	−0.28	-	1.92	2
H-2	−0.90	0.39	0.81	−0.29	7	Y-2	−0.28	1.91	-	0.13	8
H-3	0.04	1.48	0.92	0.39	6	Y-3	2.34	−0.59	-	1.50	5
H-4	−0.87	−0.09	−0.37	−0.51	9	Y-4	−2.66	−0.25	-	−1.87	13
H-5	−1.55	0.35	1.45	−0.54	10	Y-5	−2.11	−1.06	-	−1.63	12
H-6	0.33	1.17	1.38	0.55	5	Y-6	1.82	2.11	-	1.60	4
H-7	−1.06	−0.71	0.17	−0.64	13	Y-7	−1.94	−0.52	-	−1.42	11
H-8	−0.84	−0.69	−0.17	−0.57	11	Y-8	−1.61	−1.53	-	−1.36	10
H-9	−1.10	0.31	0.31	−0.47	8	Y-9	−1.66	−0.51	-	−1.22	9
H-10	1.56	−0.62	2.13	0.99	4	Y-10	0.23	2.18	-	0.53	7
H-11	4.63	−3.23	−0.23	1.83	2	Y-11	5.09	−0.56	-	3.40	1
H-12	3.87	1.04	−2.00	1.90	1	Y-12	1.20	0.50	-	0.91	6
H-13	−2.16	1.14	−2.09	−1.21	14	Y-13	−2.95	−1.10	-	−2.21	15
H-14	3.20	1.40	−0.39	1.83	2	Y-14	−3.51	1.34	-	−2.18	14
H-15	−4.22	−2.29	−0.88	−2.67	15	Y-15	3.16	−1.64	-	1.89	3
C-1	1.81	-	-	1.33	2	J-1	−1.36	1.62	0.81	−0.53	6
C-2	0.64	-	-	0.47	7	J-2	−1.57	−0.23	−0.87	−1.12	15
C-3	−0.83	-	-	−0.61	13	J-3	−1.24	2.07	1.09	−0.36	5
C-4	0.17	-	-	0.13	10	J-4	−1.17	0.41	0.20	−0.66	8
C-5	1.00	-	-	0.74	3	J-5	−1.20	0.00	−0.50	−0.81	11
C-6	0.94	-	-	0.70	4	J-6	−1.42	0.84	−1.01	−0.88	13
C-7	−0.34	-	-	−0.25	11	J-7	−1.15	−0.66	0.12	−0.81	9
C-8	0.41	-	-	0.30	8	J-8	−1.18	−1.16	0.79	−0.82	12
C-9	0.81	-	-	0.60	6	J-9	−0.71	−1.36	−0.60	−0.71	10
C-10	0.28	-	-	0.21	9	J-10	4.94	−0.39	−0.21	3.03	2
C-11	−5.01	-	-	−3.70	15	J-11	5.54	−0.29	1.24	3.58	1
C-12	0.86	-	-	0.64	5	J-12	−0.55	−1.78	−0.21	−0.63	7
C-13	1.87	-	-	1.38	1	J-13	−1.17	−0.69	−0.62	−0.90	14
C-14	−2.08	-	-	−1.54	14	J-14	−0.62	−0.04	1.76	−0.20	4
C-15	−0.55	-	-	−0.40	12	J-15	2.85	1.66	−1.98	1.81	3

**Table 2:** Three-factor Box–Behnken design (BBD) with factorial levels shown in actual values for the YQD.

Run	Independent Variables			Responses			OD(Y)
	X_1_	X_2_	X_3_	CAGC	CAC	TSR	
1	8(0)	60(1)	40(1)	0.107	0.385	0.3850	0.968
2	8(0)	45(0)	30(0)	0.087	0.341	0.3634	0.887
3	9(1)	45(0)	40(1)	0.110	0.414	0.3902	0.988
4	9(1)	30(−1)	30(0)	0.098	0.332	0.3930	0.937
5	8(0)	45(0)	30(0)	0.091	0.322	0.3816	0.907
6	7(−1)	30(−1)	30(0)	0.065	0.287	0.3342	0.000
7	7(−1)	45(0)	40(1)	0.076	0.245	0.3330	0.633
8	8(0)	60(1)	20(−1)	0.081	0.301	0.3712	0.856
9	9(1)	45(0)	20(−1)	0.098	0.337	0.3814	0.929
10	8(0)	45(0)	30(0)	0.086	0.264	0.3722	0.825
11	8(0)	30(−1)	20(−1)	0.071	0.265	0.3334	0.670
12	9(1)	60(1)	30(0)	0.099	0.371	0.4042	0.967
13	8(0)	45(0)	30(0)	0.081	0.319	0.3418	0.809
14	8(0)	30(−1)	40(1)	0.078	0.343	0.3654	0.864
15	7(−1)	60(1)	30(0)	0.069	0.254	0.3312	0.606
16	8(0)	45(0)	30(0)	0.086	0.317	0.3688	0.878
17	7(−1)	45(0)	20(−1)	0.087	0.239	0.3292	0.000

X_1_: first liquid–solid ratio (mL/g); X_2_: first extraction time (min); X_3_: second extraction time (min). CAGC: calycosin-7-*O*-β-d-glucoside content (mg/g); CAC: chlorogenic acid content (mg/g); TSR: total solid ratio (g); OD: overall desirability.

**Table 3:** Characterization of chemical constituents of YQD by UPLC-Q-TOF/MS.

ID	RT (Min)	Adduction	Measured Mass	Calculated Mass	ppm	Formula	Name	MS/MS (*m*/*z*)	Source	Type
C1	0.99	[M + H]^+^	118.0858	118.0863	−4.23	C_5_H_11_NO_2_	betaine	118.0862; 58.0644	HQ	alkaloid
C2	1.16	[M + H]^+^	144.1014	144.1019	−3.47	C_7_H_13_NO_2_	stachydrine	144.1014; 84.0801; 58.0645	CWZ	alkaloid
C3	18.55	[M − H]^−^	431.1208	431.1195	3.02	C_18_H_24_O_12_	asperulosidic acid	431.1207; 137.0244; 93.0354	HQ	iridoid glycosides
C4	18.96	[M − H]^−^	313.0923	313.0929	−1.92	C_14_H_18_O_8_	glucovanillin	151.0409; 123.0446	HQ	glycosides
C5	19.49	[M − H]^−^	353.0890	353.0878	3.40	C_16_H_18_O_9_	neochlorogenic acid	353.0900; 191.0574; 179.0356; 135.0459	CWZ	organic acids
C6	20.56	[M − H]^−^	593.2094	593.2087	1.18	C_25_H_38_O_16_	leonuriside B	593.2054; 461.1655; 135.0437	CWZ	phenylethanoid glycosides
C7	23.06	[M − H]^−^	353.0884	353.0878	1.70	C_16_H_18_O_9_	chlorogenic acid	191.0562; 85.0292	CWZ	organic acids
C8	23.74	[M − H]^−^	353.0881	353.0878	0.85	C_16_H_18_O_9_	cryptochlorogenic acid	353.0915; 191.0567; 179.0364; 173.0469; 135.0455	CWZ	organic acids
C9	25.53	[M − H]^−^	463.1254	463.1246	1.73	C_22_H_24_O_11_	hesperetin 7-*O*-glucoside	463.1243; 301.0800; 257.0800	CWZ	flavonoids-glycosides
C10	28.62	[M + H]^+^	312.1540	312.1554	−4.48	C_14_H_21_N_3_O_5_	leonurine	312.1588; 181.0482; 114.0997	CWZ	alkaloid
C11	30.41	[M − H]^−^	301.0715	301.0718	−1.00	C_16_H_14_O_6_	carasinaurone	301.0723; 257.0778; 139.00404; 124.0175	JQG	flavonoids
C12	30.86	[M + H]^+^	447.1269	447.1286	−3.80	C_22_H_22_O_10_	calycosin-7-*O*-β-d-glucoside	285.0750; 270.0517; 253.0507; 242.0541; 225.0522; 137.0217	HQ	flavonoids-glycosides
C13	33.14	[M − H]^−^	477.1419	477.1402	3.56	C_23_H_26_O_11_	calceolarioside B	477.1409; 315.1073; 161.0253; 133.0296	YZZ	phenylethanoid glycosides
C14	34.08	[M − H]^−^	515.1211	515.1195	3.11	C_25_H_24_O_12_	isochlorogenic acid B	515.1205; 353.0871; 191.0561; 173.0452; 135.0451	CWZ	organic acids
C15	34.81	[M − H]^−^	515.1212	515.1195	3.30	C_25_H_24_O_12_	isochlorogenic acid A	515.1207; 353.0871; 191.0565; 179.0363; 135.0456	CWZ	organic acids
C16	35.70	[M − H]^−^	461.1461	461.1453	1.73	C_23_H_26_O_10_	methylnissolin-3-*O*-glucoside	461.1452; 179.0371; 161.0245; 133.0303	HQ	flavonoids-glycosides
C17	36.68	[M − H]^−^	515.1202	515.1195	1.36	C_25_H_24_O_12_	isochlorogenic acid C	515.1195; 353.0877; 191.0558; 173.0460; 135.0456	CWZ	organic acids
C18	38.03	[M + FA − H]^−^	475.1247	475.1246	0.21	C_22_H_22_O_9_	ononin	267.0691; 252.0458	CWZ/HQ	flavonoids-glycosides
C19	39.17	[M + H]^+^	1075.5305	1075.532	−1.21	C_52_H_82_O_23_	mutongsaponin B	943.4894; 797.4332; 635.3815;	YZZ	triterpenoid saponins
C20	40.41	[M + FA − H]^−^	987.4837	987.4806	3.14	C_47_H_74_O_19_	saponin Ph	987.4972; 941.4768; 469.1586	YZZ	triterpenoid saponins
C21	42.03	[M − H]^−^	283.0617	283.0612	1.77	C_16_H_12_O_5_	genkwanin	283.0595; 268.0382; 211.0388; 195.0456	CWZ	flavonoids
C22	42.08	[M − H]^−^	463.1608	463.161	−0.43	C_23_H_28_O_10_	astraisoflavan-7-*O*-β-d-glucoside	463.1615; 301.1061; 179.0690	HQ	flavonoids-glycosides
C23	42.61	[M + FA − H]^−^	1003.5116	1003.5119	−0.30	C_48_H_78_O_19_	scheffoleoside A	1003.5117; 957.4984; 487.3411; 469.1534	YZZ	triterpenoid saponins
C24	42.79	[M + H]^+^	1091.5630	1091.5633	−0.27	C_53_H_86_O_23_	akebia saponin	959.5186; 813.4651; 651.4084; 489.3562	YZZ	triterpenoid saponins
C25	43.60	[M − H]^−^	431.1001	431.0984	3.94	C_21_H_20_O_10_	apigenin 7-*O*-glucoside	431.0965; 269.0419	HQ	flavonoids-glycosides
C26	43.96	[M + FA − H]^−^	1003.5109	1003.5119	−1.00	C_48_H_78_O_19_	asiaticoside	1003.5229; 957.5041; 487.3487; 469.1570	YZZ	triterpenoid saponins
C27	44.10	[M + H]^+^	1091.5579	1091.5633	−4.95	C_53_H_86_O_23_	23-hydroxyakemisaponin A	959.5174; 813.4715; 651.3946; 489.3537	YZZ	triterpenoid saponins
C28	44.73	[M + FA − H]^−^	1003.5154	1003.5119	3.49	C_48_H_78_O_19_	(2α,3β,6β)-2,3,6-trihydroxy-Olean-12-en-28-oic acid-*O*-6-deoxy-α-L-mannopyranosyl-(1→4)-*O*-β-d-glucopyranosyl-(1→6)-β-d-glucopyranosyl ester	1003.5123; 957.5028; 487.3442; 469.1551	YZZ	triterpenoid saponins
C29	45.53	[M + FA − H]^−^	969.2802	969.2764	3.92	C_56_H_44_O_13_	kobophenol A	923.2608; 801.2261	JQG	diphenylethenes
C30	49.65	[M + FA − H]^−^	1105.5449	1105.5436	1.18	C_52_H_84_O_22_	akebia saponin E	1105.5482; 1059.5363; 735.4314	YZZ	triterpenoid saponins
C31	50.55	[M + FA − H]^−^	973.5012	973.5014	−0.21	C_47_H_76_O_18_	akebia saponin D	973.4997; 927.4915; 603.3873; 323.0960; 179.0557	YZZ	triterpenoid saponins
C32	51.88	[M − H]^−^	267.0656	267.0663	−2.62	C_16_H_12_O_4_	formononetin	267.0668; 252.0436; 195.0444; 135.0122	CWZ/HQ	flavonoids
C33	55.19	[M + Cl]^−^	933.4640	933.4620	2.14	C_46_H_74_O_17_	(3β,4α)-3-[(*O*-α-L-arabinopyranosyl-(1→2)-*O*-[β-d-glucopyranosyl-(1→4)]-α-L-arabinopyranosyl)oxy]-23-hydroxy-Olean-12-en-28-oic acid	933.4631; 735.4207	YZZ	triterpenoid saponins
C34	56.36	[M + FA − H]^−^	1089.5523	1089.5487	3.30	C_52_H_84_O_21_	yuzhizioside IV	1089.5504; 1043.5381; 719.4348; 323.0966	YZZ	triterpenoid saponins
C35	59.25	[M + FA − H]^−^	871.4718	871.4697	2.41	C_43_H_70_O_15_	astragaloside II	871.4730; 825.4697; 765.4370	HQ	triterpenoid saponins
C36	61.63	[M + FA − H]^−^	871.4689	871.4697	−0.92	C_43_H_70_O_15_	astrasieversianin VII	871.4678; 825.4468	HQ	triterpenoid saponins
C37	63.61	[M + FA − H]^−^	871.4731	871.4697	3.90	C_43_H_70_O_15_	cyclosiversioside D	871.4665; 825.4716; 765.4327	HQ	triterpenoid saponins
C38	63.95	[M + FA − H]^−^	957.5079	957.5123	−4.60	C_47_H_76_O_17_	pulsatilla Saponin D	911.4976; 749.4245	YZZ	triterpenoid saponins
C39	64.27	[M − H]^−^	487.3428	487.3429	−0.21	C_30_H_48_O_5_	arjunolic acid	487.3422; 363.0096; 322.9947	YZZ	triterpenoids
C40	64.84	[M + FA − H]^−^	781.4380	781.4380	0.00	C_40_H_64_O_12_	akebia saponin B	781.4406; 735.4318; 603.3912	YZZ	triterpenoid saponins
C41	65.12	[M + FA − H]^−^	913.4847	913.4802	4.93	C_45_H_72_O_16_	astragaloside I	913.4814; 867.4702; 765.4267	HQ	triterpenoid saponins
C42	65.53	[M − H]^−^	269.0457	269.0455	0.74	C_15_H_10_O_5_	apigenin	269.0430; 225.0556	HQ	flavonoids
C43	65.71	[M + FA − H]^−^	913.4814	913.4802	1.31	C_45_H_72_O_16_	isoastragaloside I	913.4861; 867.4913	HQ	triterpenoid saponins
C44	66.41	[M + FA − H]^−^	913.4811	913.4802	0.99	C_45_H_72_O_16_	neoastragaloside I	/	HQ	triterpenoid saponins

**Table 4:** Identification of absorbed prototype components and metabolites in rat plasma by UPLC-Q-TOF/MS (1 h after the last administration).

ID	RT (Min)	Adduction	Measured Mass	Calculated Mass	ppm	Formula	Name	MS/MS (*m*/*z*)
P1(C2)	1.4	[M + H]^+^	144.1015	144.1019	−2.78	C_7_H_13_NO_2_	stachydrine	144.1019; 84.0803; 58.0649
P2(C11)	30.8	[M − H]^−^	301.0727	301.0718	2.99	C_16_H_14_O_6_	carasinaurone	273.0734; 241.0522; 193.09778; 139.0410; 124.0178
P3(C12)	31.58	[M − H]^−^	447.1281	447.1286	−1.12	C_22_H_22_O_10_	calycosin-7-*O*-β-d-glucoside	447.1179; 285.0760; 270.0554; 253.0519
P4(C40)	65.48	[M + FA − H]^−^	781.4405	781.4380	3.20	C_40_H_64_O_12_	akebia saponin B	781.4385; 735.4349; 603.4002; 471.3462
M1	25.78	[M − H]^−^	509.0412	509.0395	3.34	C_21_H_18_O_13_S	daidzein + sulfation + glucuronidation	509.0401; 333.0079; 253.0511
M2	27.92	[M − H]^−^	621.1487	621.1461	4.19	C_28_H_30_O_16_	calycosin-7-*O*-β-d-glucoside + glucuronidation	621.1492; 459.0964; 283.0620; 268.0344
M3	28.14	[M − H]^−^	509.0415	509.0395	3.93	C_21_H_18_O_13_S	daidzein + sulfation + glucuronidation	509.0419; 429.0845; 333.0045; 253.0527
M4	28.81	[M − H]^−^	429.0836	429.0827	2.10	C_21_H_18_O_10_	daidzein + glucuronidation	253.0516; 175.0232
M5	28.81	[M − H]^−^	525.0359	525.0345	2.67	C_21_H_18_O_14_S	apigenin + sulfation + glucuronidation	525.0370; 349.0028; 269.0465
M6	29.71	[M − H]^−^	459.0946	459.0933	2.83	C_22_H_20_O_11_	genkwanin + glucuronidation	459.0964; 283.0616; 268.0389; 240.0447
M7	30.75	[M − H]^−^	497.0775	497.0759	3.22	C_21_H_22_O_12_S	equol + sulfation + glucuronidation	497.0759; 417.1175; 321.0430; 241.0857; 175.0232
M8	31.56	[M − H]^−^	429.0843	429.0827	3.73	C_21_H_18_O_10_	daidzein + glucuronidation	429.0854; 253.0514; 129.0216; 113.0250
M9	31.75	[M − H]^−^	541.0301	541.0294	1.29	C_21_H_18_O_15_S	apigenin + hydroxylation + glucuronidation + sulfation	541.0318; 461.0719; 285.0413
M10	32.96	[M − H]^−^	445.0794	445.0776	4.04	C_21_H_18_O_11_	apigenin + glucuronidation	445.0820; 269.0482; 224.0483; 175.0253; 113.0251
M11	33.69	[M − H]^−^	433.1158	433.114	4.16	C_21_H_22_O_10_	2′,4′,7-trihydroxyisoflavan + glucuronidation	433.12775; 296.9257; 257.0741; 175.0232; 135.0435
M12	33.65	[M − H]^−^	491.1575	491.1559	3.26	C_24_H_28_O_11_	7-hydroxy-2′,3′,4′-trimethoxyisoflavan + glucuronidation	491.1598; 315.1253; 271.1348
M13	34.78	[M − H]^−^	445.0798	445.0776	4.94	C_21_H_18_O_11_	apigenin + glucuronidation	445.2026; 269.0479; 180.0724
M14	34.84	[M − H]^−^	333.0089	333.0074	4.50	C_15_H_10_O_7_S	daidzein + sulfation	333.0095; 253.0519; 224.0475; 117.0347
M15	34.88	[M − H]^−^	447.0940	447.0933	1.57	C_21_H_20_O_11_	7,3′,4′-trihydroxyisoflavanone + glucuronidation	447.0942; 271.0625; 198.9095; 165.0206
M16	35.55	[M − H]^−^	459.0950	459.0933	3.70	C_22_H_20_O_11_	calycosin + glucuronidation	459.0962; 283.0612; 268.0391; 175.0251
M17	35.55	[M − H]^−^	417.1207	417.1191	3.84	C_21_H_22_O_9_	equol + glucuronidation	417.1223; 399.1180; 241.0886; 175.0258
M18	35.91	[M − H]^−^	447.0943	447.0933	2.24	C_21_H_20_O_11_	7,3′,4′-trihydroxyisoflavanone + glucuronidation	447.1322; 271.0639; 151.0070; 113.0255
M19	36.18	[M − H]^−^	417.1210	417.1191	4.56	C_21_H_22_O_9_	equol + glucuronidation	417.1217; 399.1116; 241.0871; 175.0269
M20	36.11	[M − H]^−^	639.1957	639.1931	4.07	C_29_H_36_O_16_	astraisoflavan-7-*O*-β-d-glucoside + glucuronidation	639.1943; 463.1845; 301.1089; 286.0901
M21	38.58	[M − H]^−^	417.1207	417.1191	3.84	C_21_H_22_O_9_	equol + glucuronidation	417.1214; 241.0878; 175.0247; 121.0303
M22	38.63	[M − H]^−^	321.0449	321.0438	3.43	C_15_H_14_O_6_S	equol + sulfation	321.0489; 242.0913; 121.0305
M23	38.92	[M − H]^−^	443.0999	443.0984	3.39	C_22_H_20_O_10_	formononetin + glucuronidation	443.1701; 267.0670; 252.0441; 223.0398; 179.0250
M24	40.04	[M − H]^−^	253.0511	253.0506	1.98	C_15_H_10_O_4_	formononetin + demethylation	253.0518; 225.0558; 135.0094
M25	40.86	[M − H]^−^	433.1153	433.114	3.00	C_21_H_22_O_10_	2′,4′,7-trihydroxyisoflavan + glucuronidation	433.2095; 257.0832; 175.0253; 113.0252
M26	41.42	[M − H]^−^	433.1157	433.114	3.93	C_21_H_22_O_10_	2′,4′,7-trihydroxyisoflavan + glucuronidation	433.1166; 257.0826; 136.0182
M27	41.99	[M − H]^−^	475.1267	475.1246	4.42	C_23_H_24_O_11_	methylnissolin + glucuronidation	475.1302; 301.1060; 284.0711; 269.0862
M28	42.76	[M − H]^−^	459.0951	459.0933	3.92	C_22_H_20_O_11_	calycosin + glucuronidation	459.0979; 283.0662; 268.0368; 255.0686
M29	43.13	[M − H]^−^	477.1421	477.1402	3.98	C_23_H_26_O_11_	astraisoflavan + glucuronidation	477.1445; 301.1100; 286.0859; 271.0616
M30	43.28	[M + H]^+^	303.1212	303.1227	−4.95	C_17_H_18_O_5_	astraisoflavan	303.1024; 167.0699; 133.0677; 123.0434
M31	44.51	[M − H]^−^	445.1154	445.114	3.15	C_22_H_22_O_10_	formononetin + hydrogenation + glucuronidation	445.1165; 269.0850; 254.0608; 217.0108; 113.0268
M32	45.68	[M − H]^−^	347.0230	347.0231	−0.29	C_16_H_12_O_7_S	formononetin + sulfation	347.0279; 267.0650; 252.0431; 223.0376

**Table 5:** Information table on active components of YQD.

ID	Name	Formula	MW
C13	Calceolarioside B	C_23_H_26_O_11_	478.15
C24	Akebia saponin PJ1	C_53_H_86_O_23_	1090.56
C30	Akebia saponin E	C_52_H_84_O_22_	1060.54
C31	Akebia saponin D	C_47_H_76_O_18_	928.50
C42	Apigenin	C_15_H_10_O_5_	270.05
P1	Stachydrine	C_7_H_13_NO_2_	143.09
P2	Carasinaurone	C_16_H_14_O_6_	302.08
P3	Calycosin-7-*O*-β-d-glucoside	C_22_H_22_O_10_	446.12
P4	Akebia saponin B	C_40_H_64_O_12_	736.44
M2	Calycosin-7-*O*-β-d-glucoside+glucuronidation	C_28_H_30_O_16_	622.15
M5	Apigenin+sulfation+glucuronidation	C_21_H_18_O_14_S	526.04
M6	Genkwanin+glucuronidation	C_22_H_20_O_11_	460.10
M9	Apigenin+hydroxylation+glucuronidation+sulfation	C_21_H_18_O_15_S	542.04
M10	Apigenin+glucuronidation	C_21_H_18_O_11_	446.08
M11	2′,4′,7-trihydroxyisoflavan+glucuronidation	C_21_H_22_O_10_	434.12
M12	7-hydroxy-2′,3′,4′-trimethoxyisoflavan+glucuronidation	C_24_H_28_O_11_	492.16
M13	Apigenin+glucuronidation	C_21_H_18_O_11_	446.08
M15	7,3′,4′-trihydroxyisoflavanone+glucuronidation	C_21_H_20_O_11_	448.10
M16	Calycosin+glucuronidation	C_22_H_20_O_11_	460.10
M18	7,3′,4′-trihydroxyisoflavanone+glucuronidation	C_21_H_20_O_11_	448.10
M20	Astraisoflavan-7-*O*-β-d-glucoside+glucuronidation	C_29_H_36_O_16_	640.20
M23	Formononetin+glucuronidation	C_22_H_20_O_10_	444.11
M24	Formononetin+demethylation	C_15_H_10_O_4_	254.06
M25	2′,4′,7-trihydroxyisoflavan+glucuronidation	C_21_H_22_O_10_	434.12
M26	2′,4′,7-trihydroxyisoflavan+glucuronidation	C_21_H_22_O_10_	434.12
M27	Methylnissolin+glucuronidation	C_23_H_24_O_11_	476.13
M28	Calycosin+glucuronidation	C_22_H_20_O_11_	460.10
M29	Astraisoflavan+glucuronidation	C_23_H_26_O_11_	478.15
M30	Astraisoflavan	C_17_H_18_O_5_	302.12
M31	Formononetin+hydrogenation+glucuronidation	C_22_H_22_O_10_	446.12
M32	Formononetin+sulfation	C_16_H_12_O_7_S	348.03

## Data Availability

The data used to support the findings of this study are included in the article.

## Supplementary Materials

The following supporting information can be downloaded at: https://www.mdpi.com/article/10.3390/ph16121716/s1. Tables S1: Similarity analysis of the fingerprint chromatograms; Table S2: Eigenvalue and variance contribution rate; Table S3: ANOVA table for the OD (Box-Behnken design); Table S4: Experimental validation results; Table S5: The content of CAG and CA in 10 batches of YQD; Table S6: Compounds and target used for network pharmacology analysis; Table S7: Overlapping of drug-related targets and disease-related targets; Table S8: Data of PPI network diagrams; Table S9: The degree value in the CTD network; Table S10: Data of KEGG analysis; Table S11: The degree value in the CTP network; Table S12: Activation rate of YQD to mushroom tyrosinase. Table S13: The toxicity of YQD to zebrafish embryos; Table S14: Origin and batch information of HQ, YZZ, CWZ, and JQG. Figure S1: Loading plot of HQ (A); YZZ (B); CWZ (C); and JQG (D); Figure S2: The interaction surface (A) and the contour diagram (B) of the first extraction time (X_2_) and the second extraction time (X_3_); Figure S3: (A) The condition of zebrafish 72 hpf in the presence of YQD; (B) The calculated LC_50_ of YQD in zebrafish.

## Abbreviations

YQD	Yiqi Qubai standard decoction
TCM	Traditional Chinese medicine
HCA	Hierarchical cluster analysis
PCA	Principal component analysis
RSM	Response surface methodology
BBD	Box–Behnken design
UPLC-Q-TOF/MS	Ultrahigh performance liquid chromatography coupled with quadrupole-time of flight mass spectrometry
HPLC	High performance liquid chromatography
SPE	Solid-phase extraction
CTD	Compound–target–disease
PPI	Protein–protein interaction
CTP	Component–target–pathway
KEGG	Kyoto Encyclopedia of Genes and Genomes
GO	Gene Ontology
mTOR	Mammalian target of rapamycin
MAPK1	Mitogen-activated protein kinase 1
PI3K	Phosphoinositide 3-kinase
AKT	Serine-threonine kinase
FoxO	Forkhead box, class O
TNF	Tumor necrosis factor
MITF	Microphthalmia-associated transcription factor
TYR	Tyrosinase
TRP-1	Tyrosinase-related proteins 1
TRP-2	Tyrosinase-related proteins 2
