# Supplementary material for: A Systematic Study of Yiqi Qubai Standard Decoction for Treating Vitiligo Based on UPLC-Q-TOF/MS Combined with Chemometrics, Molecular Docking, and Cellular and Zebrafish Assays
Source: Pharmaceuticals (Basel). 2023 Dec 11;16(12):1716. doi: 10.3390/ph16121716 (PMC10747336; doi:10.3390/ph16121716)
Supplement: Supplementary file 1 [file pharmaceuticals-16-01716-s001.zip › Table S1 Similarity analysis of the fingerprint chromatograms.pdf]

Table S1-1 Similarity analysis of HQ

| | H-1 | H-2 | H-3 | H-4 | H-5 | H-6 | H-7 | H-8 | H-9 | H-10 | H-11 | H-12 | H-13 | H-14 | H-15 | R |
|---|---|---|---|---|---|---|---|---|---|---|---|---|---|---|---|---|
| H-1 | 1 | 0.993 | 0.998 | 0.995 | 0.972 | 0.989 | 0.976 | 0.989 | 0.992 | 0.975 | 0.982 | 0.983 | 0.988 | 0.991 | 0.952 | 0.994 |
| H-2 | 0.993 | 1 | 0.998 | 0.995 | 0.99 | 0.995 | 0.991 | 0.997 | 0.999 | 0.987 | 0.981 | 0.977 | 0.981 | 0.986 | 0.976 | 0.998 |
| H-3 | 0.998 | 0.998 | 1 | 0.998 | 0.984 | 0.995 | 0.986 | 0.995 | 0.997 | 0.983 | 0.982 | 0.978 | 0.984 | 0.988 | 0.968 | 0.997 |
| H-4 | 0.995 | 0.995 | 0.998 | 1 | 0.986 | 0.998 | 0.989 | 0.993 | 0.996 | 0.99 | 0.987 | 0.978 | 0.975 | 0.987 | 0.964 | 0.997 |
| H-5 | 0.972 | 0.99 | 0.984 | 0.986 | 1 | 0.992 | 0.998 | 0.991 | 0.991 | 0.994 | 0.968 | 0.951 | 0.949 | 0.964 | 0.987 | 0.988 |
| H-6 | 0.989 | 0.995 | 0.995 | 0.998 | 0.992 | 1 | 0.994 | 0.993 | 0.997 | 0.995 | 0.986 | 0.973 | 0.968 | 0.983 | 0.97 | 0.997 |
| H-7 | 0.976 | 0.991 | 0.986 | 0.989 | 0.998 | 0.994 | 1 | 0.993 | 0.993 | 0.997 | 0.976 | 0.96 | 0.953 | 0.971 | 0.984 | 0.992 |
| H-8 | 0.989 | 0.997 | 0.995 | 0.993 | 0.991 | 0.993 | 0.993 | 1 | 0.997 | 0.988 | 0.979 | 0.972 | 0.976 | 0.982 | 0.984 | 0.996 |
| H-9 | 0.992 | 0.999 | 0.997 | 0.996 | 0.991 | 0.997 | 0.993 | 0.997 | 1 | 0.989 | 0.985 | 0.98 | 0.98 | 0.989 | 0.975 | 0.999 |
| H-10 | 0.975 | 0.987 | 0.983 | 0.99 | 0.994 | 0.995 | 0.997 | 0.988 | 0.989 | 1 | 0.984 | 0.962 | 0.947 | 0.973 | 0.971 | 0.991 |
| H-11 | 0.982 | 0.981 | 0.982 | 0.987 | 0.968 | 0.986 | 0.976 | 0.979 | 0.985 | 0.984 | 1 | 0.99 | 0.965 | 0.993 | 0.935 | 0.991 |
| H-12 | 0.983 | 0.977 | 0.978 | 0.978 | 0.951 | 0.973 | 0.96 | 0.972 | 0.98 | 0.962 | 0.99 | 1 | 0.983 | 0.998 | 0.919 | 0.985 |
| H-13 | 0.988 | 0.981 | 0.984 | 0.975 | 0.949 | 0.968 | 0.953 | 0.976 | 0.98 | 0.947 | 0.965 | 0.983 | 1 | 0.987 | 0.936 | 0.98 |
| H-14 | 0.991 | 0.986 | 0.988 | 0.987 | 0.964 | 0.983 | 0.971 | 0.982 | 0.989 | 0.973 | 0.993 | 0.998 | 0.987 | 1 | 0.936 | 0.993 |
| H-15 | 0.952 | 0.976 | 0.968 | 0.964 | 0.987 | 0.97 | 0.984 | 0.984 | 0.975 | 0.971 | 0.935 | 0.919 | 0.936 | 0.936 | 1 | 0.968 |
| R | 0.994 | 0.998 | 0.997 | 0.997 | 0.988 | 0.997 | 0.992 | 0.996 | 0.999 | 0.991 | 0.991 | 0.985 | 0.98 | 0.993 | 0.968 | 1 |

Table S1-2 Similarity analysis of YZZ

| | Y-1 | Y-2 | Y-3 | Y-4 | Y-5 | Y-6 | Y-7 | Y-8 | Y-9 | Y-10 | Y-11 | Y-12 | Y-13 | Y-14 | Y-15 | R |
|---|---|---|---|---|---|---|---|---|---|---|---|---|---|---|---|---|
| Y-1 | 1 | 0.988 | 0.992 | 0.872 | 0.873 | 0.997 | 0.92 | 0.929 | 0.949 | 0.989 | 0.997 | 0.979 | 0.786 | 0.886 | 0.987 | 0.978 |
| Y-2 | 0.988 | 1 | 0.982 | 0.923 | 0.923 | 0.991 | 0.959 | 0.95 | 0.97 | 0.985 | 0.988 | 0.996 | 0.854 | 0.935 | 0.975 | 0.993 |
| Y-3 | 0.992 | 0.982 | 1 | 0.896 | 0.898 | 0.993 | 0.932 | 0.956 | 0.969 | 0.984 | 0.986 | 0.978 | 0.807 | 0.905 | 0.993 | 0.985 |
| Y-4 | 0.872 | 0.923 | 0.896 | 1 | 0.997 | 0.881 | 0.99 | 0.97 | 0.969 | 0.882 | 0.872 | 0.943 | 0.966 | 0.981 | 0.894 | 0.954 |
| Y-5 | 0.873 | 0.923 | 0.898 | 0.997 | 1 | 0.884 | 0.99 | 0.963 | 0.964 | 0.874 | 0.87 | 0.946 | 0.976 | 0.989 | 0.89 | 0.954 |
| Y-6 | 0.997 | 0.991 | 0.993 | 0.881 | 0.884 | 1 | 0.924 | 0.933 | 0.954 | 0.988 | 0.991 | 0.981 | 0.798 | 0.9 | 0.984 | 0.981 |
| Y-7 | 0.92 | 0.959 | 0.932 | 0.99 | 0.99 | 0.924 | 1 | 0.976 | 0.981 | 0.922 | 0.922 | 0.974 | 0.952 | 0.982 | 0.929 | 0.98 |
| Y-8 | 0.929 | 0.95 | 0.956 | 0.97 | 0.963 | 0.933 | 0.976 | 1 | 0.997 | 0.944 | 0.931 | 0.958 | 0.885 | 0.944 | 0.959 | 0.978 |
| Y-9 | 0.949 | 0.97 | 0.969 | 0.969 | 0.964 | 0.954 | 0.981 | 0.997 | 1 | 0.963 | 0.951 | 0.976 | 0.887 | 0.952 | 0.97 | 0.99 |
| Y-10 | 0.989 | 0.985 | 0.984 | 0.882 | 0.874 | 0.988 | 0.922 | 0.944 | 0.963 | 1 | 0.992 | 0.972 | 0.773 | 0.88 | 0.986 | 0.976 |
| Y-11 | 0.997 | 0.988 | 0.986 | 0.872 | 0.87 | 0.991 | 0.922 | 0.931 | 0.951 | 0.992 | 1 | 0.979 | 0.782 | 0.883 | 0.983 | 0.977 |
| Y-12 | 0.979 | 0.996 | 0.978 | 0.943 | 0.946 | 0.981 | 0.974 | 0.958 | 0.976 | 0.972 | 0.979 | 1 | 0.89 | 0.959 | 0.968 | 0.996 |
| Y-13 | 0.786 | 0.854 | 0.807 | 0.966 | 0.976 | 0.798 | 0.952 | 0.885 | 0.887 | 0.773 | 0.782 | 0.89 | 1 | 0.976 | 0.789 | 0.887 |
| Y-14 | 0.886 | 0.935 | 0.905 | 0.981 | 0.989 | 0.9 | 0.982 | 0.944 | 0.952 | 0.88 | 0.883 | 0.959 | 0.976 | 1 | 0.886 | 0.957 |
| Y-15 | 0.987 | 0.975 | 0.993 | 0.894 | 0.89 | 0.984 | 0.929 | 0.959 | 0.97 | 0.986 | 0.983 | 0.968 | 0.789 | 0.886 | 1 | 0.98 |
| R | 0.978 | 0.993 | 0.985 | 0.954 | 0.954 | 0.981 | 0.98 | 0.978 | 0.99 | 0.976 | 0.977 | 0.996 | 0.887 | 0.957 | 0.98 | 1 |

Table S1-3 Similarity analysis of CWZ

| | C-1 | C-2 | C-3 | C-4 | C-5 | C-6 | C-7 | C-8 | C-9 | C-10 | C-11 | C-12 | C-13 | C-14 | C-15 | R |
|---|---|---|---|---|---|---|---|---|---|---|---|---|---|---|---|---|
| C-1 | 1 | 0.981 | 0.994 | 0.993 | 0.999 | 0.978 | 0.987 | 0.991 | 0.989 | 0.988 | 0.986 | 0.993 | 0.976 | 0.961 | 0.988 | 0.994 |
| C-2 | 0.981 | 1 | 0.989 | 0.994 | 0.987 | 0.984 | 0.999 | 0.967 | 0.992 | 0.987 | 0.958 | 0.976 | 0.998 | 0.961 | 0.997 | 0.993 |
| C-3 | 0.994 | 0.989 | 1 | 0.99 | 0.997 | 0.969 | 0.992 | 0.981 | 0.984 | 0.98 | 0.99 | 0.993 | 0.979 | 0.962 | 0.987 | 0.992 |
| C-4 | 0.993 | 0.994 | 0.99 | 1 | 0.995 | 0.994 | 0.998 | 0.988 | 0.999 | 0.998 | 0.965 | 0.989 | 0.994 | 0.975 | 0.999 | 1 |

| | | | | | | | | | | | | | | | | |
|---|---|---|---|---|---|---|---|---|---|---|---|---|---|---|---|---|
| C-5 | 0.999 | 0.987 | 0.997 | 0.995 | 1 | 0.98 | 0.992 | 0.99 | 0.991 | 0.989 | 0.985 | 0.994 | 0.982 | 0.964 | 0.991 | 0.997 |
| C-6 | 0.978 | 0.984 | 0.969 | 0.994 | 0.98 | 1 | 0.988 | 0.98 | 0.997 | 0.999 | 0.933 | 0.972 | 0.991 | 0.974 | 0.994 | 0.992 |
| C-7 | 0.987 | 0.999 | 0.992 | 0.998 | 0.992 | 0.988 | 1 | 0.978 | 0.996 | 0.992 | 0.964 | 0.985 | 0.997 | 0.972 | 0.998 | 0.997 |
| C-8 | 0.991 | 0.967 | 0.981 | 0.988 | 0.99 | 0.98 | 0.978 | 1 | 0.986 | 0.988 | 0.97 | 0.995 | 0.965 | 0.982 | 0.978 | 0.99 |
| C-9 | 0.989 | 0.992 | 0.984 | 0.999 | 0.991 | 0.997 | 0.996 | 0.986 | 1 | 0.999 | 0.955 | 0.984 | 0.995 | 0.975 | 0.999 | 0.998 |
| C-10 | 0.988 | 0.987 | 0.98 | 0.998 | 0.989 | 0.999 | 0.992 | 0.988 | 0.999 | 1 | 0.95 | 0.982 | 0.991 | 0.976 | 0.996 | 0.997 |
| C-11 | 0.986 | 0.958 | 0.99 | 0.965 | 0.985 | 0.933 | 0.964 | 0.97 | 0.955 | 0.95 | 1 | 0.985 | 0.942 | 0.932 | 0.957 | 0.97 |
| C-12 | 0.993 | 0.976 | 0.993 | 0.989 | 0.994 | 0.972 | 0.985 | 0.995 | 0.984 | 0.982 | 0.985 | 1 | 0.969 | 0.98 | 0.981 | 0.992 |
| C-13 | 0.976 | 0.998 | 0.979 | 0.994 | 0.982 | 0.991 | 0.997 | 0.965 | 0.995 | 0.991 | 0.942 | 0.969 | 1 | 0.962 | 0.998 | 0.992 |
| C-14 | 0.961 | 0.961 | 0.962 | 0.975 | 0.964 | 0.974 | 0.972 | 0.982 | 0.975 | 0.976 | 0.932 | 0.98 | 0.962 | 1 | 0.967 | 0.977 |
| C-15 | 0.988 | 0.997 | 0.987 | 0.999 | 0.991 | 0.994 | 0.998 | 0.978 | 0.999 | 0.996 | 0.957 | 0.981 | 0.998 | 0.967 | 1 | 0.998 |
| R | 0.994 | 0.993 | 0.992 | 1 | 0.997 | 0.992 | 0.997 | 0.99 | 0.998 | 0.997 | 0.97 | 0.992 | 0.992 | 0.977 | 0.998 | 1 |

Table S1-4 Similarity analysis of JQG

| | J-1 | J-2 | J-3 | J-4 | J-5 | J-6 | J-7 | J-8 | J-9 | J-10 | J-11 | J-12 | J-13 | J-14 | J-15 | R |
|---|---|---|---|---|---|---|---|---|---|---|---|---|---|---|---|---|
| J-1 | 1 | 0.989 | 0.998 | 0.999 | 0.999 | 0.999 | 0.986 | 0.984 | 0.995 | 0.904 | 0.911 | 0.991 | 0.993 | 0.917 | 0.993 | 0.987 |
| J-2 | 0.989 | 1 | 0.988 | 0.99 | 0.993 | 0.993 | 0.98 | 0.978 | 0.98 | 0.894 | 0.898 | 0.979 | 0.987 | 0.912 | 0.983 | 0.979 |
| J-3 | 0.998 | 0.988 | 1 | 0.997 | 0.997 | 0.998 | 0.979 | 0.977 | 0.991 | 0.91 | 0.918 | 0.987 | 0.99 | 0.922 | 0.989 | 0.988 |
| J-4 | 0.999 | 0.99 | 0.997 | 1 | 0.999 | 0.998 | 0.99 | 0.989 | 0.997 | 0.91 | 0.916 | 0.995 | 0.996 | 0.924 | 0.996 | 0.991 |
| J-5 | 0.999 | 0.993 | 0.997 | 0.999 | 1 | 0.999 | 0.989 | 0.987 | 0.995 | 0.906 | 0.912 | 0.993 | 0.995 | 0.921 | 0.995 | 0.989 |
| J-6 | 0.999 | 0.993 | 0.998 | 0.998 | 0.999 | 1 | 0.985 | 0.983 | 0.993 | 0.899 | 0.907 | 0.989 | 0.993 | 0.916 | 0.992 | 0.986 |
| J-7 | 0.986 | 0.98 | 0.979 | 0.99 | 0.989 | 0.985 | 1 | 0.999 | 0.992 | 0.885 | 0.89 | 0.995 | 0.997 | 0.905 | 0.997 | 0.979 |
| J-8 | 0.984 | 0.978 | 0.977 | 0.989 | 0.987 | 0.983 | 0.999 | 1 | 0.991 | 0.883 | 0.887 | 0.994 | 0.996 | 0.9 | 0.996 | 0.977 |
| J-9 | 0.995 | 0.98 | 0.991 | 0.997 | 0.995 | 0.993 | 0.992 | 0.991 | 1 | 0.906 | 0.913 | 0.999 | 0.997 | 0.919 | 0.999 | 0.988 |
| J-10 | 0.904 | 0.894 | 0.91 | 0.91 | 0.906 | 0.899 | 0.885 | 0.883 | 0.906 | 1 | 0.999 | 0.901 | 0.905 | 0.995 | 0.896 | 0.957 |
| J-11 | 0.911 | 0.898 | 0.918 | 0.916 | 0.912 | 0.907 | 0.89 | 0.887 | 0.913 | 0.999 | 1 | 0.908 | 0.91 | 0.994 | 0.903 | 0.961 |
| J-12 | 0.991 | 0.979 | 0.987 | 0.995 | 0.993 | 0.989 | 0.995 | 0.994 | 0.999 | 0.901 | 0.908 | 1 | 0.997 | 0.915 | 0.999 | 0.986 |
| J-13 | 0.993 | 0.987 | 0.99 | 0.996 | 0.995 | 0.993 | 0.997 | 0.996 | 0.997 | 0.905 | 0.91 | 0.997 | 1 | 0.922 | 0.999 | 0.989 |
| J-14 | 0.917 | 0.912 | 0.922 | 0.924 | 0.921 | 0.916 | 0.905 | 0.9 | 0.919 | 0.995 | 0.994 | 0.915 | 0.922 | 1 | 0.912 | 0.966 |
| J-15 | 0.993 | 0.983 | 0.989 | 0.996 | 0.995 | 0.992 | 0.997 | 0.996 | 0.999 | 0.896 | 0.903 | 0.999 | 0.999 | 0.912 | 1 | 0.985 |
| R | 0.987 | 0.979 | 0.988 | 0.991 | 0.989 | 0.986 | 0.979 | 0.977 | 0.988 | 0.957 | 0.961 | 0.986 | 0.989 | 0.966 | 0.985 | 1 |
