# Supplementary material for: A Systematic Study of Yiqi Qubai Standard Decoction for Treating Vitiligo Based on UPLC-Q-TOF/MS Combined with Chemometrics, Molecular Docking, and Cellular and Zebrafish Assays
Source: Pharmaceuticals (Basel). 2023 Dec 11;16(12):1716. doi: 10.3390/ph16121716 (PMC10747336; doi:10.3390/ph16121716)
Supplement: Supplementary file 1 [file pharmaceuticals-16-01716-s001.zip › Table S12 Activation rate of YQD to mushroom tyrosinase.pdf]

Table S12. Activation rate of YQD to mushroom tyrosinase

Concentration（mg/mL)	Activation rate（%）
0.25	28.38
0.5	31.74
1	35.46
1.5	45.00
2	49.16
4	52.34
6	62.51
8	79.66
