# Supplementary material for: A Systematic Study of Yiqi Qubai Standard Decoction for Treating Vitiligo Based on UPLC-Q-TOF/MS Combined with Chemometrics, Molecular Docking, and Cellular and Zebrafish Assays
Source: Pharmaceuticals (Basel). 2023 Dec 11;16(12):1716. doi: 10.3390/ph16121716 (PMC10747336; doi:10.3390/ph16121716)
Supplement: Supplementary file 1 [file pharmaceuticals-16-01716-s001.zip › Table S13 The toxicity of YQD to zebrafish embryos.pdf]

Table S13. The toxicity of YQD to zebrafish embryos

Dose (μg/mL)	pericardium edema rate (%)		yolk absorption delay rate (%)		mortality rate (%)	
	Group 1	Group 2	Group 1	Group 2	Group 1	Group 2
0	0	0	0	0	3.3	3.3
100	0	0	0	0	3.3	10
500	0	0	0	0	3.3	10
1000	0	0	0	0	0	3.3
2000	36.7	/	33.3	/	50	100
4000	/	/	/	/	100	100
