# Supplementary material for: A Systematic Study of Yiqi Qubai Standard Decoction for Treating Vitiligo Based on UPLC-Q-TOF/MS Combined with Chemometrics, Molecular Docking, and Cellular and Zebrafish Assays
Source: Pharmaceuticals (Basel). 2023 Dec 11;16(12):1716. doi: 10.3390/ph16121716 (PMC10747336; doi:10.3390/ph16121716)
Supplement: Supplementary file 1 [file pharmaceuticals-16-01716-s001.zip › Table S14 Origin and batch information of HQ, YZZ, CWZ, and JQG.pdf]

Table S14. Origin and batch information of HQ, YZZ, CWZ, and JQG

Name	Sample No.	Batch No.	Origins	Name	Sample No.	Batch No.	Origins
HQ	H1	20200702	Dingxi, Gansu	YZZ	Y1	20180801	Nanyang, Henan
HQ	H2	20200401	Dingxi, Gansu	YZZ	Y2	20190402	Nanyang, Henan
HQ	H3	20200802	Dingxi, Gansu	YZZ	Y3	20200101	Nanyang, Henan
HQ	H4	20210402	Dingxi, Gansu	YZZ	Y4	20190821	Nanyang, Henan
HQ	H5	20210502	Dingxi, Gansu	YZZ	Y5	20180701	Nanyang, Henan
HQ	H6	20210201	Dingxi, Gansu	YZZ	Y6	20181002	Nanyang, Henan
HQ	H7	20210401	Dingxi, Gansu	YZZ	Y7	20201224	Jingmen, Hubei
HQ	H8	20201001	Dingxi, Gansu	YZZ	Y8	20200801	Jingmen, Hubei
HQ	H9	20200901	Dingxi, Gansu	YZZ	Y9	20190901	Jingmen, Hubei
HQ	H10	20210302	Dingxi, Gansu	YZZ	Y10	20200715	Jingmen, Hubei
HQ	H11	20201008	Zhangye, Gansu	YZZ	Y11	20200728	Yichang, Hubei
HQ	H12	20200312	Zhangye, Gansu	YZZ	Y12	20200401	Badong, Hubei
HQ	H13	20210418	Neimeng	YZZ	Y13	20190401	Anhui
HQ	H14	20210102	Neimeng	YZZ	Y14	20190501	Anhui
HQ	H15	20210302	Shanxi	YZZ	Y15	20190801	Anhui
CWZ	C1	20210501	Nanyang, Henan	JQG	J1	20200801	Jingmen, Hubei
CWZ	C2	20200601	Nanyang, Henan	JQG	J2	20200602	Jingmen, Hubei
CWZ	C3	20200701	Nanyang, Henan	JQG	J3	20201001	Jingmen, Hubei
CWZ	C4	20201201	Nanyang, Henan	JQG	J4	20200401	Jingmen, Hubei
CWZ	C5	20200301	Nanyang, Henan	JQG	J5	20200301	Jingmen, Hubei
CWZ	C6	20200402	Nanyang, Henan	JQG	J6	20200402	Jingmen, Hubei
CWZ	C7	20190501	Nanyang, Henan	JQG	J7	20190601	Jingmen, Hubei
CWZ	C8	20200801	Nanyang, Henan	JQG	J8	20190201	Jingmen, Hubei
CWZ	C9	20190201	Nanyang, Henan	JQG	J9	20190502	Jingmen, Hubei
CWZ	C10	20200801	Nanyang, Henan	JQG	J10	20191019	Yichang, Hubei
CWZ	C11	20200523	Nanyang, Henan	JQG	J11	20190927	Yichang, Hubei
CWZ	C12	20201019	Chifeng, Neimeng	JQG	J12	20200401	Xiangyang, Hubei

CWZ	C13	20190201	Chifeng, Neimeng	JQG	J13	20200301	Xiangyang, Hubei
CWZ	C14	20200901	Anhui	JQG	J14	20190901	Yuanan, Hubei
CWZ	C15	20200701	Fuxin, Liaoning	JQG	J15	20190801	Sichuan
