# Supplementary material for: A Systematic Study of Yiqi Qubai Standard Decoction for Treating Vitiligo Based on UPLC-Q-TOF/MS Combined with Chemometrics, Molecular Docking, and Cellular and Zebrafish Assays
Source: Pharmaceuticals (Basel). 2023 Dec 11;16(12):1716. doi: 10.3390/ph16121716 (PMC10747336; doi:10.3390/ph16121716)
Supplement: Supplementary file 1 [file pharmaceuticals-16-01716-s001.zip › Table S2 Eigenvalue and variance contribution rate.pdf]

Table S2-1 Eigenvalue and variance contribution rate of HQ

Peak no.	Initial eigenvalue			Extracing eigenvalue		
	Total	Variance contribution rate %	Cumulative variance contribution rate %	Total	Variance contribution rate %	Cumulative variance contribution rate %
1	5.676	51.601	51.601	5.676	51.601	51.601
2	1.817	16.515	68.116	1.817	16.515	68.116
3	1.484	13.493	81.609	1.484	13.493	81.609
4	0.882	8.015	89.624			
5	0.549	4.99	94.614			
6	0.341	3.103	97.717			
7	0.176	1.602	99.319			
8	0.047	0.43	99.749			
9	0.021	0.192	99.941			
10	0.005	0.047	99.988			
11	0.001	0.012	100			

Table S2-2 Eigenvalue and variance contribution rate of YZZ

Peak no.	Initial eigenvalue			Extracing eigenvalue		
	Total	Variance contribution rate %	Cumulative variance contribution rate %	Total	Variance contribution rate %	Cumulative variance contribution rate %
1	6.864	68.637	68.637	6.864	68.637	68.637
2	1.683	16.83	85.467	1.683	16.83	85.467
3	0.65	6.5	91.967			
4	0.432	4.321	96.288			
5	0.186	1.865	98.153			
6	0.113	1.131	99.283			
7	0.043	0.429	99.712			
8	0.017	0.172	99.884			
9	0.01	0.099	99.983			
10	0.002	0.017	100			

Table S2-3 Eigenvalue and variance contribution rate of CWZ

Peak no.	Initial eigenvalue			Extracing eigenvalue		
	Total	Variance contribution rate %	Cumulative variance contribution rate %	Total	Variance contribution rate %	Cumulative variance contribution rate %
1	2.952	73.803	73.803	2.952	73.803	73.803
2	0.717	17.933	91.736			
3	0.243	6.075	97.811			

4	0.088	2.189	100

Table S2-4 Eigenvalue and variance contribution rate of JQG

Peak no.	Initial eigenvalue			Extracing eigenvalue		
	Total	Variance contribution rate %	Cumulative variance contribution rate %	Total	Variance contribution rate %	Cumulative variance contribution rate %
1	5.656	62.842	62.842	5.656	62.842	62.842
2	1.291	14.347	77.189	1.291	14.347	77.189
3	0.995	11.053	88.242	0.995	11.053	88.242
4	0.57	6.33	94.572			
5	0.327	3.63	98.203			
6	0.084	0.928	99.131			
7	0.053	0.588	99.719			
8	0.024	0.271	99.989			
9	0.001	0.011	100			
