# Supplementary material for: A Systematic Study of Yiqi Qubai Standard Decoction for Treating Vitiligo Based on UPLC-Q-TOF/MS Combined with Chemometrics, Molecular Docking, and Cellular and Zebrafish Assays
Source: Pharmaceuticals (Basel). 2023 Dec 11;16(12):1716. doi: 10.3390/ph16121716 (PMC10747336; doi:10.3390/ph16121716)
Supplement: Supplementary file 1 [file pharmaceuticals-16-01716-s001.zip › Table S3 ANOVA table for the OD (Box-Behnken design).pdf]

Table S3 ANOVA table for the OD (Box-Behnken design)

| Source | Sum of squares | df | *F* value | *P* value |
|---|---|---|---|---|
| Model | 1.44 | 9 | 27.46 | 0.0001 |
| *$X_1$ | 0.8333 | 1 | 143.35 | ＜0.0001 |
| *$X_2$ | 0.1072 | 1 | 18.44 | 0.0036 |
| *$X_3$ | 0.1245 | 1 | 21.42 | 0.0024 |
| $X_1X_2$ | 0.0829 | 1 | 14.27 | 0.0069 |
| $X_1X_3$ | 0.0824 | 1 | 14.17 | 0.0070 |
| $X_2X_3$ | 0.0017 | 1 | 0.2892 | 0.6074 |
| $X_1^2$ | 0.1998 | 1 | 34.37 | 0.0006 |
| $X_2^2$ | 0.0011 | 1 | 0.1820 | 0.6825 |
| $X_3^2$ | 0.0001 | 1 | 0.0248 | 0.8793 |
| Residual | 0.0407 | 7 | | |
| Lack of Fit | 0.0336 | 3 | 6.33 | 0.0534 |
| Pure Error | 0.0071 | 4 | | |
| Cor Total | 1.48 | 16 | | |

*$X_1$: First liquid- solid ratio (mL/g)

*$X_2$: First extraction time (min)

*$X_3$: Second extraction time (min)
