# Supplementary material for: A Systematic Study of Yiqi Qubai Standard Decoction for Treating Vitiligo Based on UPLC-Q-TOF/MS Combined with Chemometrics, Molecular Docking, and Cellular and Zebrafish Assays
Source: Pharmaceuticals (Basel). 2023 Dec 11;16(12):1716. doi: 10.3390/ph16121716 (PMC10747336; doi:10.3390/ph16121716)
Supplement: Supplementary file 1 [file pharmaceuticals-16-01716-s001.zip › Table S4 Experimental validation results.pdf]

Table S4 Experimental validation results

Run	Calycosin-7-O-β-D-glucoside (mg/g)	Chlorogenic acid (mg/g)	Total solid ratio (g)	OD (Y)
1	0.108	0.312	0.4032	0
2	0.109	0.356	0.384	0.959
3	0.099	0.343	0.3878	0
4	0.106	0.326	0.3508	0
5	0.11	0.334	0.4126	0.960
6	0.107	0.354	0.3962	0.961
RSD（%）	3.69	5.03	5.50	0.13
