# Supplementary material for: A Systematic Study of Yiqi Qubai Standard Decoction for Treating Vitiligo Based on UPLC-Q-TOF/MS Combined with Chemometrics, Molecular Docking, and Cellular and Zebrafish Assays
Source: Pharmaceuticals (Basel). 2023 Dec 11;16(12):1716. doi: 10.3390/ph16121716 (PMC10747336; doi:10.3390/ph16121716)
Supplement: Supplementary file 1 [file pharmaceuticals-16-01716-s001.zip › Table S5 The content of CAG and CA in 10 batches of YQD.pdf]

Table S5. The content of CAG and CA in 10 batches of YQD

Samples	Batch No.				Calycosin-7-O-β-D-glucoside (mg/g)	Chlorogenic acid (mg/g)
	HQ	YZZ	CWZ	JQG		
1	20200301	20200101	20200401	20200302	0.116	0.308
2	20200101	20200301	20191201	20200801	0.095	0.304
3	20190502	20200501	20200301	20200401	0.086	0.275
4	20190403	20190502	20200601	20200301	0.106	0.374
5	20190803	20200602	20200701	20190502	0.088	0.383
6	20200601	20190801	20190501	20190402	0.111	0.268
7	20200401	20200401	20190201	20200201	0.105	0.299
8	20190201	20190301	20200101	20190801	0.097	0.334
9	20200102	20191101	20190401	20190901	0.111	0.334
10	20191102	20200801	20200201	20190201	0.125	0.376
RSD (%)					11.99	12.82
