# Supplementary figures and images for: A Systematic Study of Yiqi Qubai Standard Decoction for Treating Vitiligo Based on UPLC-Q-TOF/MS Combined with Chemometrics, Molecular Docking, and Cellular and Zebrafish Assays
Source: Pharmaceuticals (Basel). 2023 Dec 11;16(12):1716. doi: 10.3390/ph16121716 (PMC10747336; doi:10.3390/ph16121716)

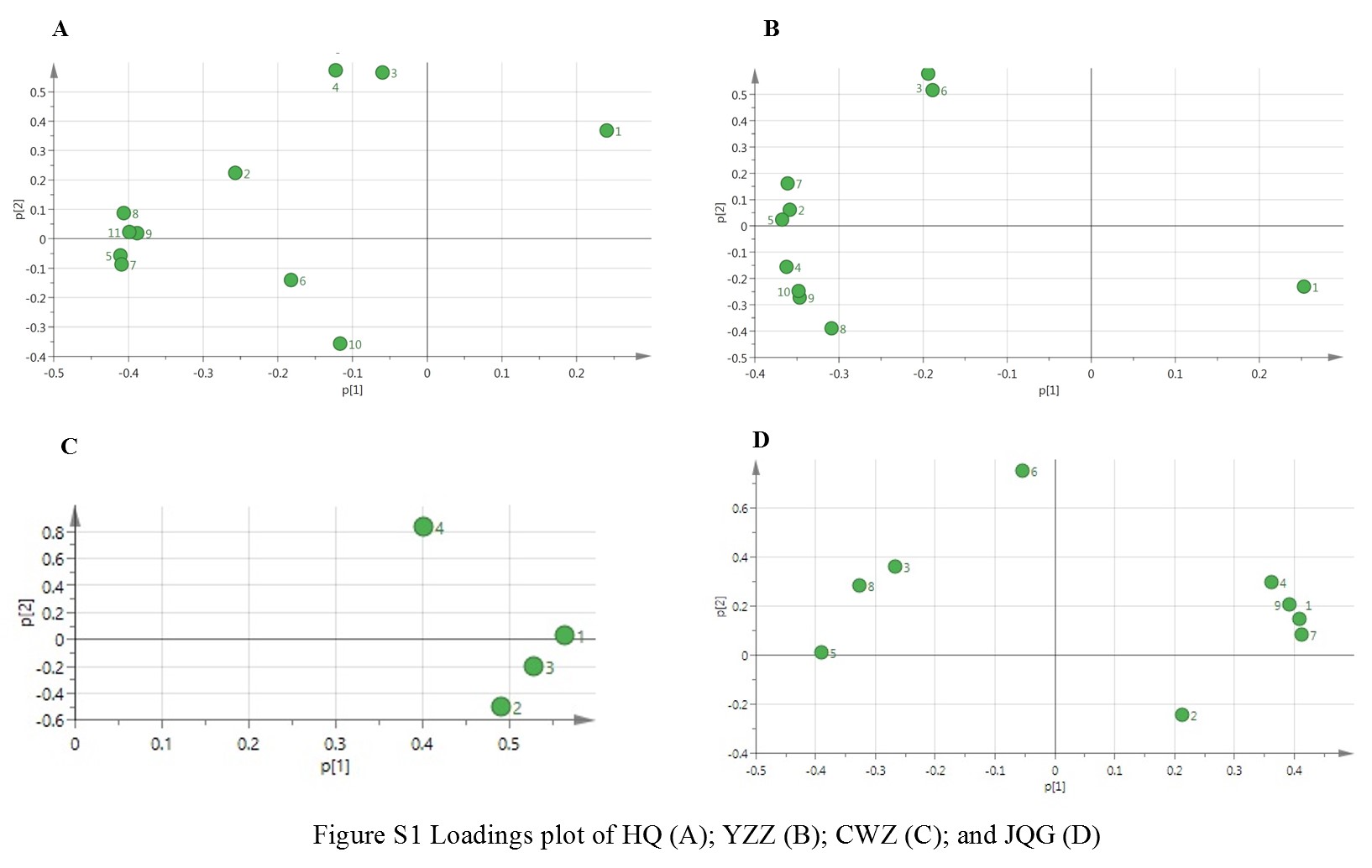

Supplement: Supplementary file 1 [file pharmaceuticals-16-01716-s001.zip › Figure S1 Loading plot of HQ (A); YZZ (B); CWZ (C); and JQG (D).jpg]

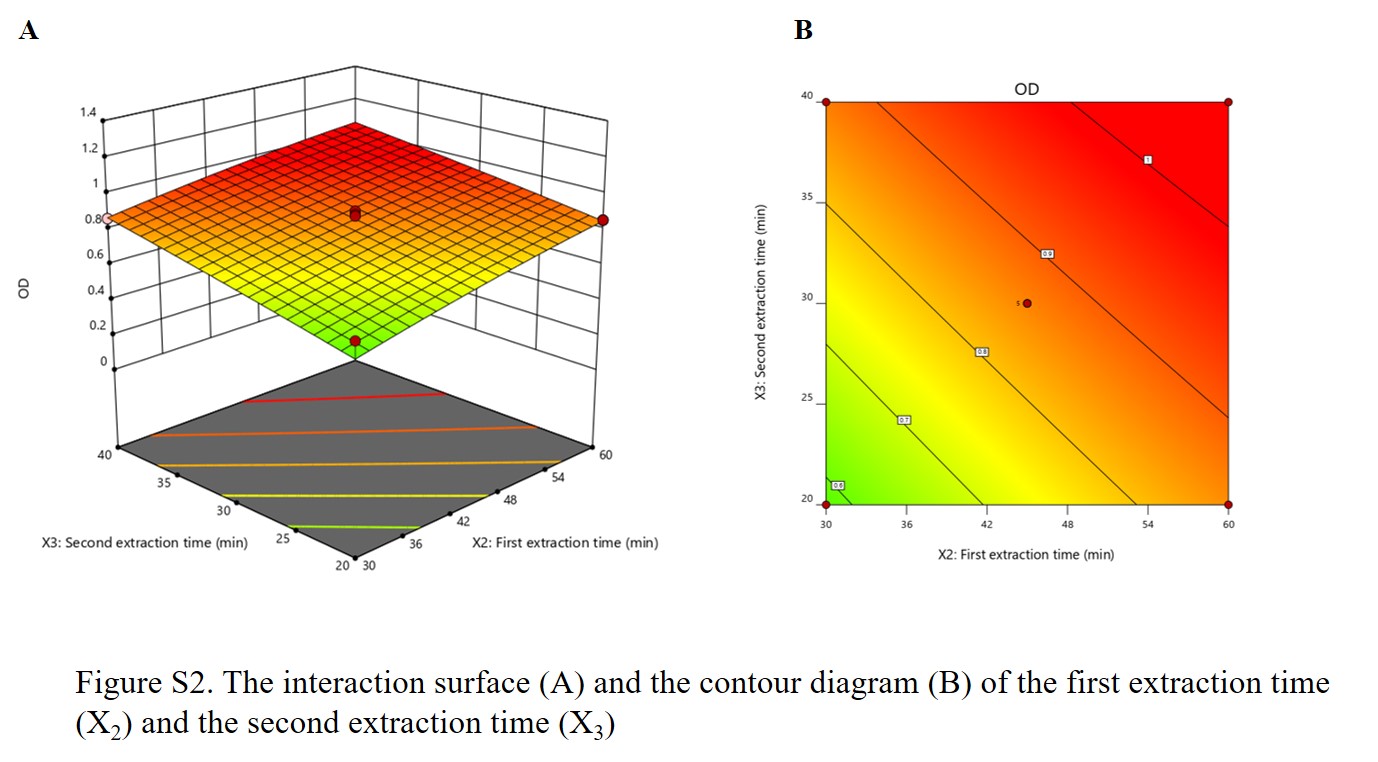

Supplement: Supplementary file 1 [file pharmaceuticals-16-01716-s001.zip › Figure S2 The interaction surface (A) and the contour diagram (B) of the first extraction time (X2) and the second extraction time (X3).jpg]

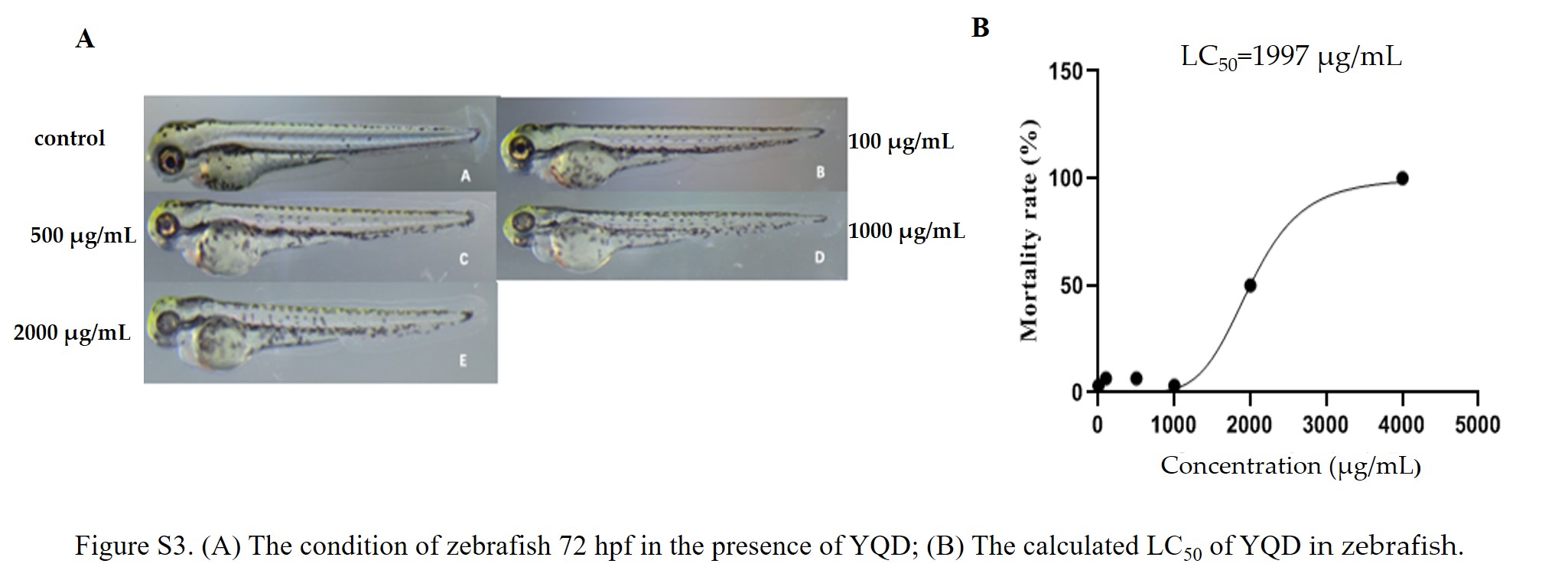

Supplement: Supplementary file 1 [file pharmaceuticals-16-01716-s001.zip › Figure S3 (A) The condition of zebrafish 72 hpf in the presence of YQD; (B) The calculated LC50 of YQD in zebrafish.jpg]
